# Phenome-wide association study on miRNA-related sequence variants: the UK Biobank

**DOI:** 10.1186/s40246-023-00553-w

**Published:** 2023-11-24

**Authors:** Rima Mustafa, Mohsen Ghanbari, Ville Karhunen, Marina Evangelou, Abbas Dehghan

**Affiliations:** 1 Department of Epidemiology and Biostatistics, Imperial College London, London, UK; 2 UK Dementia Research Institute, Imperial College London, London, UK; 3 Big Data Institute, Li Ka Shing Centre for Health Information and Discovery, University of Oxford, Oxford, UK; 4 Nuffield Department of Population Health, University of Oxford, Oxford, UK; 5 Department of Epidemiology, Erasmus MC University Medical Center, Rotterdam, The Netherlands; 6 Research Unit of Mathematical Sciences, University of Oulu, Oulu, Finland; 7 Research Unit of Population Health, University of Oulu, Oulu, Finland; 8 Department of Mathematics, Imperial College London, London, UK; 9 MRC Centre for Environment and Health, Imperial College London, London, UK

**Keywords:** microRNA, Genetic variants, Phenome, Pleiotropy

## Abstract

**Background:**

Genetic variants in the coding region could directly affect the structure and expression levels of genes and proteins. However, the importance of variants in the non-coding region, such as microRNAs (miRNAs), remain to be elucidated. Genetic variants in miRNA-related sequences could affect their biogenesis or functionality and ultimately affect disease risk. Yet, their implications and pleiotropic effects on many clinical conditions remain unknown.

**Methods:**

Here, we utilised genotyping and hospital records data in the UK Biobank (*N* = 423,419) to investigate associations between 346 genetic variants in miRNA-related sequences and a wide range of clinical diagnoses through phenome-wide association studies. Further, we tested whether changes in blood miRNA expression levels could affect disease risk through colocalisation and Mendelian randomisation analysis.

**Results:**

We identified 122 associations for six variants in the seed region of miRNAs, nine variants in the mature region of miRNAs, and 27 variants in the precursor miRNAs. These included associations with hypertension, dyslipidaemia, immune-related disorders, and others. Nineteen miRNAs were associated with multiple diagnoses, with six of them associated with multiple disease categories. The strongest association was reported between rs4285314 in the precursor of miR-3135b and celiac disease risk (odds ratio (OR) per effect allele increase = 0.37, *P* = 1.8 × 10^–162^). Colocalisation and Mendelian randomisation analysis highlighted potential causal role of miR-6891-3p in dyslipidaemia.

**Conclusions:**

Our study demonstrates the pleiotropic effect of miRNAs and offers insights to their possible clinical importance.

**Supplementary Information:**

The online version contains supplementary material available at 10.1186/s40246-023-00553-w.

## Background

MicroRNAs (miRNAs) are small non-coding ribonucleic acids (RNA) of approximately 22 nucleotides that regulate gene expression and play critical roles in determining whether genes are active and how much protein is produced [1]. Mature miRNAs can perform their functions either in the cytoplasm or be released from the cell to the circulation and body fluids [2] to serve as chemical messengers and facilitate cell-to-cell communications [3]. These miRNAs can bind to target mRNAs to suppress translation [1] through inhibiting translation or affect degradation of mRNAs [4, 5]. A single miRNA can bind to multiple target genes, leading to a complex regulatory mechanism. Conversely, each gene could be targeted by multiple miRNAs [6].

Previous studies have explored the role of miRNAs in complex disorders through various approaches, including observational and experimental studies. Experimental studies typically assess molecular changes following administration miRNA mimics or antagonists and an assessment of the expression of potential target genes [7–10]. Candidate-based studies still dominate the studies of miRNAs in complex disorders, although unbiased high-throughput approaches are increasingly reported [11, 12]. With over 2000 miRNAs currently identified in humans [13], there is an opportunity for a comprehensive investigation to identify novel candidate miRNAs in complex diseases. Among observational studies, relatively few have leveraged genetic association data for circulatory miRNAs. The use of genetic data to study miRNAs could minimise the effect of confounders and avoid reverse causation since the genetic variants are fixed during conception.

Similar to other genes, the non-coding sequences of miRNAs are also subject to genetic variation, which could exist in the seed, mature, or precursor sequences of miRNAs [14], albeit the density is lower than the other parts of human genome [15–17], indicating that these regions are evolutionary conserved. The genetic variants in the seed region could interfere with the interaction between miRNA and target mRNA and, thus, are expected to be more functional [15, 18]. Meanwhile, those residing outside the seed region can affect binding beyond the seed region and the strength of inhibition of miRNA target [19], influence the biogenesis of mature miRNAs [20], or the processing of primary miRNA to precursor miRNAs [21]. Since a single miRNA could have multiple target genes [22], genetic variants residing in the miRNA-related sequence could affect the expression of target genes and downstream biological processes.

Extensive research has investigated the associations between genetic variants in miRNA-related sequences, i.e., seed, mature, or precursor regions with specific type of disorders including cancers [23, 24] and cardiometabolic traits [24, 25]. We previously showed that pleiotropy is common for miRNAs [26], which highlights the value of investigating many phenotypes rather than a single trait or disease for each miRNA. However, no systematic investigation of genetic variants in miRNAs on a wide range of traits has been published thus far.

In this study, genetic variants residing in different regions of miRNAs were used to proxy miRNA expression levels and link them with an extensive range of clinical conditions in the UK Biobank through phenome-wide association studies (PheWAS). We further tested for the presence of an effect of miRNAs on clinical conditions through colocalisation and Mendelian randomisation analysis. This approach offered the first large-scale systematic analysis to investigate the effects of variants in miRNA sequences on human diseases in a hypothesis-free manner and discover novel associations with possible clinical importance.

## Methods

### Study population

This study used individual-level data from the UK Biobank, a large prospective cohort of ~ 500,000 participants in the UK [27]. The UK Biobank recruited individuals aged 40–69 years old living in the UK between 2006 and 2010. This cohort collects extensive phenotype and genotype data, including longitudinal follow-up provided in the hospital episode statistics (HES) data.

Participants were asked to give blood samples at enrolment from which the DNA was extracted. Genotyping was conducted at Affymetrix Research Services Laboratory. Quality control of the data was carried out at the Wellcome Trust Centre for Human Genetics [28]. Genome-wide genotyping was performed on all UK Biobank participants, covering ~ 805,000 markers. Marker-based quality control was conducted using 463,844 participants of European ancestries, where they were tested for batch effects, plate effects, Hardy–Weinberg Equilibrium (HWE), sex effects, array effects, and discordance across replicates. Any marker that failed in at least one of those tests was set to have a missing genotype call.

Imputation in the UK Biobank was conducted using UK10K and 1000 Genomes Phase 3 reference panels, consisting of 87,696,888 bi-allelic markers in 12,570 haplotypes for interim data release (~ 150,000 samples). Further, the HRC reference panel consisting of 39,235,157 markers in 64,976 haplotypes was incorporated in the remaining samples' imputation. The major histocompatibility complex (MHC) region in chromosome six was imputed separately using a specific algorithm [29]. The UCSC genome annotation database for the Genome Reference Consortium Human Build 37 (GRCh37) assembly of the human genome was used to assign the dbSNP Reference SNP (rs) IDs.

In our study, one of each pair or relatives was excluded based on a kinship coefficient of > 0.088, the proposed lower-limit kinship-coefficient threshold for second-degree relatives [30]. Analysis was restricted to participants who identified as “White”. Additionally, individuals who asked to withdraw from the cohort until the time of analysis were excluded (Additional file 1: Fig. S1).

### Selection of candidate variants

**Fig. 1:**
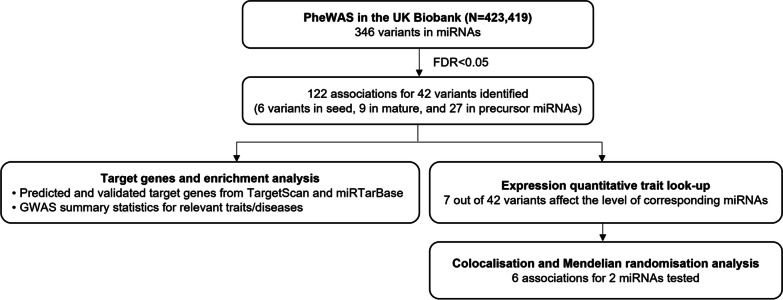
Study overview. We conducted PheWAS on variants in miRNAs using the UK Biobank data (*N* = 423,419). Putative target genes for miRNAs with FDR-significant findings in their seed regions were identified by leveraging databases for target genes of miRNAs and the genome-wide association studies (GWAS) hits for relevant traits. Further, we tested whether changes in miRNA expression could affect disease risk through colocalisation and Mendelian randomisation

An overview of our study is presented in Fig. 1. We used a database from the most recent version of miRNASNP-v3 (http://bioinfo.life.hust.edu.cn/miRNASNP/#!/) [31] to obtain the list of candidate variants residing in miRNA genes. The dataset was available from containing 46,826 unique variants residing in 1897 precursor miRNAs and corresponding to 2625 mature miRNAs.

Our analysis focused on well-imputed common variants. From the list of variants, 370 of them were available in the imputed genetic data in the UK Biobank. There were 350 variants remained after filtering for info score > 0.7 and minor allele frequency > 0.01. Multi-allelic variants were further excluded, resulting in 346 variants included in our analysis (Additional file 1: Table S1). In total, we tested for 43 variants in the seed region of 44 miRNAs, 45 variants in the mature region of 66 miRNAs, and 238 variants in 208 precursor miRNAs (Fig. 1).

### Phenome-wide association studies (PheWAS)

PheWAS was conducted separately for each candidate variant using the *PheWAS* package in R [32]. We used the hospital episode statistics data in the UK Biobank including a total of 8,404,826 episodes. There were 372,256 participants with at least one episode of diagnosis in all hospital inpatient records. ICD (ninth and tenth editions) codes from hospital episode statistics data were aligned into phecodes to identify clinically related phenotypes and assign a case–control status for each participant [33]. The analysis was conducted for phecodes with at least 200 cases [34]. Logistic regression was performed for each genetic variant with adjustment for age, sex, genotyping array, and the first five genetic principal components to account for population stratification. In this study, we accounted for multiple testing by controlling for false discovery rate (FDR), which estimates the proportion of falsely rejected hypothesis among all tests [35]. This method was chosen because hospital diagnoses are not entirely independent of each other.

### Target gene and enrichment analysis

For findings in the seed region of miRNAs, putative target genes for miRNAs that might be involved in the disease process were identified by leveraging the GWAS summary statistics for the traits of interest and target genes from TargetScan v7.2 [36] and miRTarBase [37]. Enrichment analysis for target genes were conducted for miRNAs and their counterparts following the method applied in our previous work [26].

### Colocalisation and Mendelian randomisation (MR)

Previous expression quantitative trait loci (eQTL) analysis on blood or plasma levels of miRNAs were used to check whether the genetic variants affect the level of corresponding miRNAs or acting as miRNA expression quantitative trait loci (miR-eQTLs) [38, 39]. For associations with evidence of miR-eQTLs, we performed colocalisation using summary statistics of miR-eQTLs in the Rotterdam Study [38] and summary statistics from large GWAS of the trait of interest. For each miRNA-disease pair, we used genomic region extending 200 kb on either side of mature miRNA position according to miRBase [13]. We implemented a Bayesian framework to test for the presence of shared causal variant and calculated posterior probability to declare as evidence of colocalisation implemented in *coloc* package in R [40].

Colocalisation method estimates the posterior probabilities (PP) of four scenarios/hypotheses: H0: neither trait has a genetic association in the region, H1: only trait 1 (miRNA) has a genetic association in the region, H2: only trait 2 (disease) has a genetic association in the region, H3: both traits are associated with different causal variants, H4: both traits are associated and share a single causal variant. Default priors were used in all analyses. Results with PP H4 > 0.5 were reported and those with PP H4 > 0.7 were considered significant. We also extracted SNP with the highest SNP PP H4 (posterior probability that the SNP being causal conditional on H4 being true) as the likely shared causal variant between miRNAs and the clinical conditions.

For findings with evidence of colocalisation, we further conducted MR analysis using the same genetic variants used for PheWAS and estimating the MR effect size using the Wald ratio method. The genetic association estimates between the instruments and miRNAs were taken from the Rotterdam Study [38], whereas between the instruments and clinical outcomes were taken from the UK Biobank.

## Results

### Study participants

This analysis used individual-level data from the UK Biobank, a large prospective cohort of ~ 500,000 participants in the UK [27]. After excluding related participants, those who self-identified as non-White, and withdrawn participants, 423,419 participants were included (Additional file 1: Fig. S1). Of those, 54% were female. The mean (SD) of age of participants was 56.8 (7.9) years (Table 1). The hospital-based diagnoses coded in both ICD9/10 were mapped into 1805 phecodes to enable conducting PheWAS, of which 905 phecodes had at least 200 cases across 16 disease groups (Table 2).

**Table 1:** Descriptive characteristics of the UK Biobank participants in PheWAS (*N* = 423,419)

Characteristics	Mean/*N* (SD/%)
Age, years (SD)	56.8 (7.9)
Sex, female (%)	228,441 (53.5)
BMI (SD)	27.4 (4.8)
SBP, mmHg (SD)	139.9 (19.7)
DBP, mmHg (SD)	82.2 (10.7)
History of type 2 diabetes	22,054 (5.2)
History of coronary heart disease	22,314 (5.3)
Smoking status	
Current smoker	44,061 (10.4)
Previous smoker	150,323 (35.5)
Never smoker	227,534 (53.7)
Alcohol drinking status	
Current	394,792 (93.2)
Previous	14,766 (3.5)
Never	13,477 (3.4)
Lipid	
Total cholesterol, mmol/L (SD)	5.7 (1.14)
LDL-C, mmol/L (SD)	3.6 (0.87)
HDL-C, mmol/L (SD)	1.5 (0.38)
Triglycerides, mmol/L (SD)	1.8 (1.02)
Employment status	
Employed (including self-employed)	241,669 (57.1)
Retired	144,895 (34.2)
Student (full or part-time)	874 (0.21)
Unemployed	6,061 (1.43)

*SD* standard deviation, *N* number of participants, *BMI* body mass index, *SBP* systolic blood pressure, *DBP* diastolic blood pressure, *LDL-C* low-density lipoprotein cholesterol, *HDL-C* high-density lipoprotein cholesterol

**Table 2:** Disease groups and number of cases for diagnoses with at least 200 cases in UK Biobank

Disease groups	Number of diagnoses	Number of cases			
		Minimum	Median	Mean	Maximum
Infectious diseases	27	223	1368	2001	9811
Neoplasms	83	206	899	2469	25,561
Endocrine/metabolic	69	203	827	4489	42,979
Hematopoietic	30	213	716	2089	14,597
Mental disorders	36	212	873	3064	15,848
Neurological	45	203	554	1402	13,336
Sense organs	76	203	736	1668	24,862
Circulatory system	110	215	1512	5280	91,878
Respiratory	55	217	2012	2870	12,908
Digestive	118	224	1945	5295	41,580
Genitourinary	51	235	1519	3091	16,317
Dermatologic	49	235	964	1895	8940
Musculoskeletal	72	215	1031	3874	51,437
Congenital anomalies	26	201	472	692	1960
Symptoms	16	210	2242	4863	19,563
Injuries and poisonings	42	219	933	2810	20,790

### Phenome-wide association studies

**Fig. 2:**
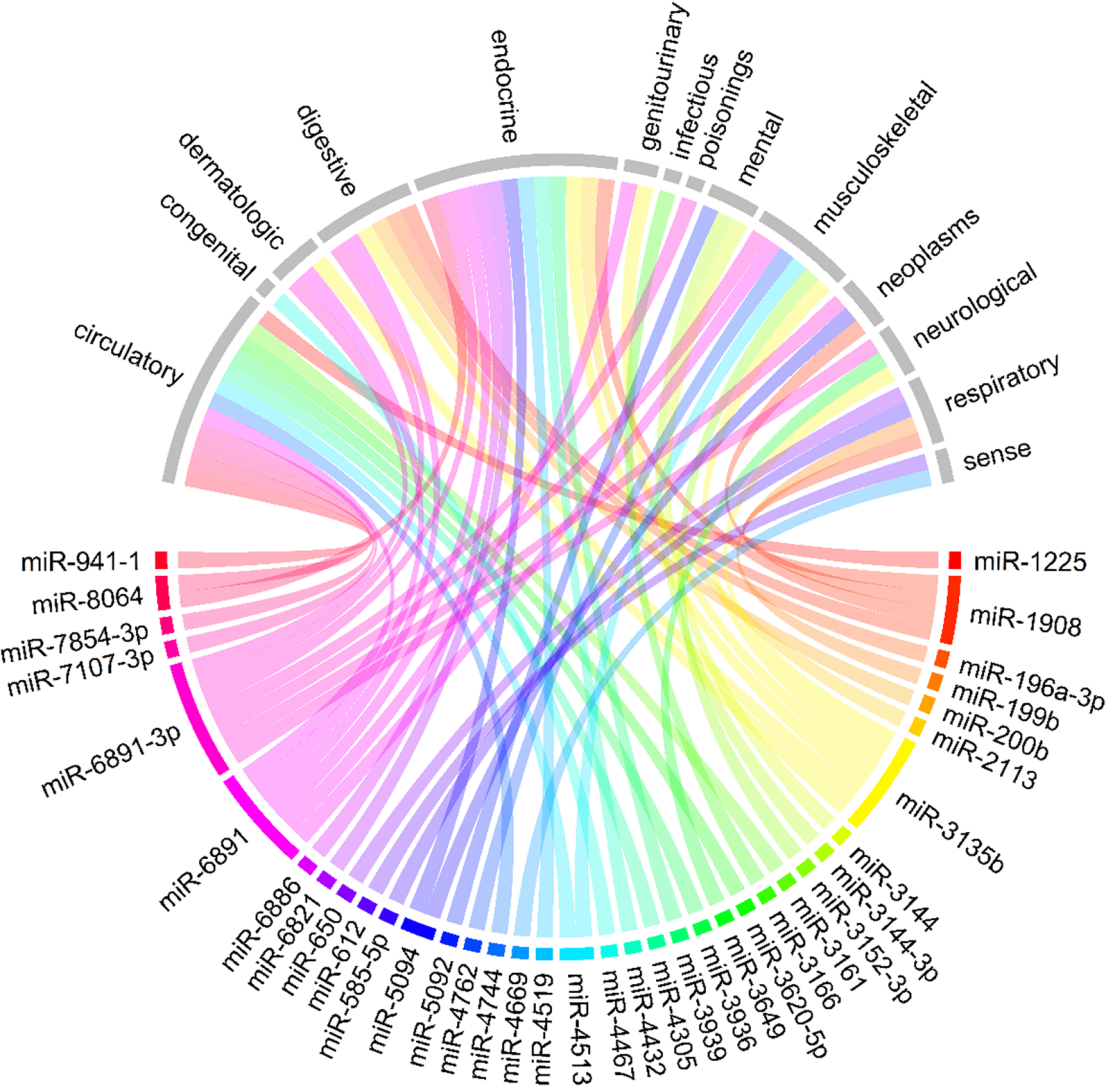
Circular plot shows the links between genetic variants in miRNAs (coloured outer circle in bottom half) and clinical diagnoses (grey outer circle in upper half). Each line represents FDR-significant association between genetic variants in miRNAs and clinical diagnoses belonging to a particular disease group

We included 346 common genetic variants in our analysis, corresponding to 43 variants in the seed region of 44 miRNAs, 45 variants in the mature region of 66 miRNAs, and 238 variants in 208 precursor miRNAs (Additional file 1: Table S1). The minor allele frequencies of tested variants ranged from 0.03 to 0.33 (in seed region), 0.01–0.42 (in mature region), 0.01–0.46 (in precursor miRNAs). PheWAS was conducted for each of those 346 variants by testing against 905 phecodes with at least 200 cases. At FDR < 0.05 (*P* < 2.15 × 10^–5^), there were 122 significant associations for 35 miRNAs, consisting of six variants in the seed region, nine variants in the mature region, and 27 variants in the precursor sequences (Fig. 2, Additional file 1: Table S2). Of 35 miRNAs with significant findings, nineteen were associated with multiple diagnoses, with six of them associated with multiple disease categories. The strongest associations were reported between rs4285314 in the precursor gene of miR-3135b and celiac disease risk (odds ratio (OR) per effect allele increase = 0.37, *P* = 1.8 × 10^–162^). The strong association found in current study highlighted the value of genetic studies using large population-based cohort.

**Fig. 3:**
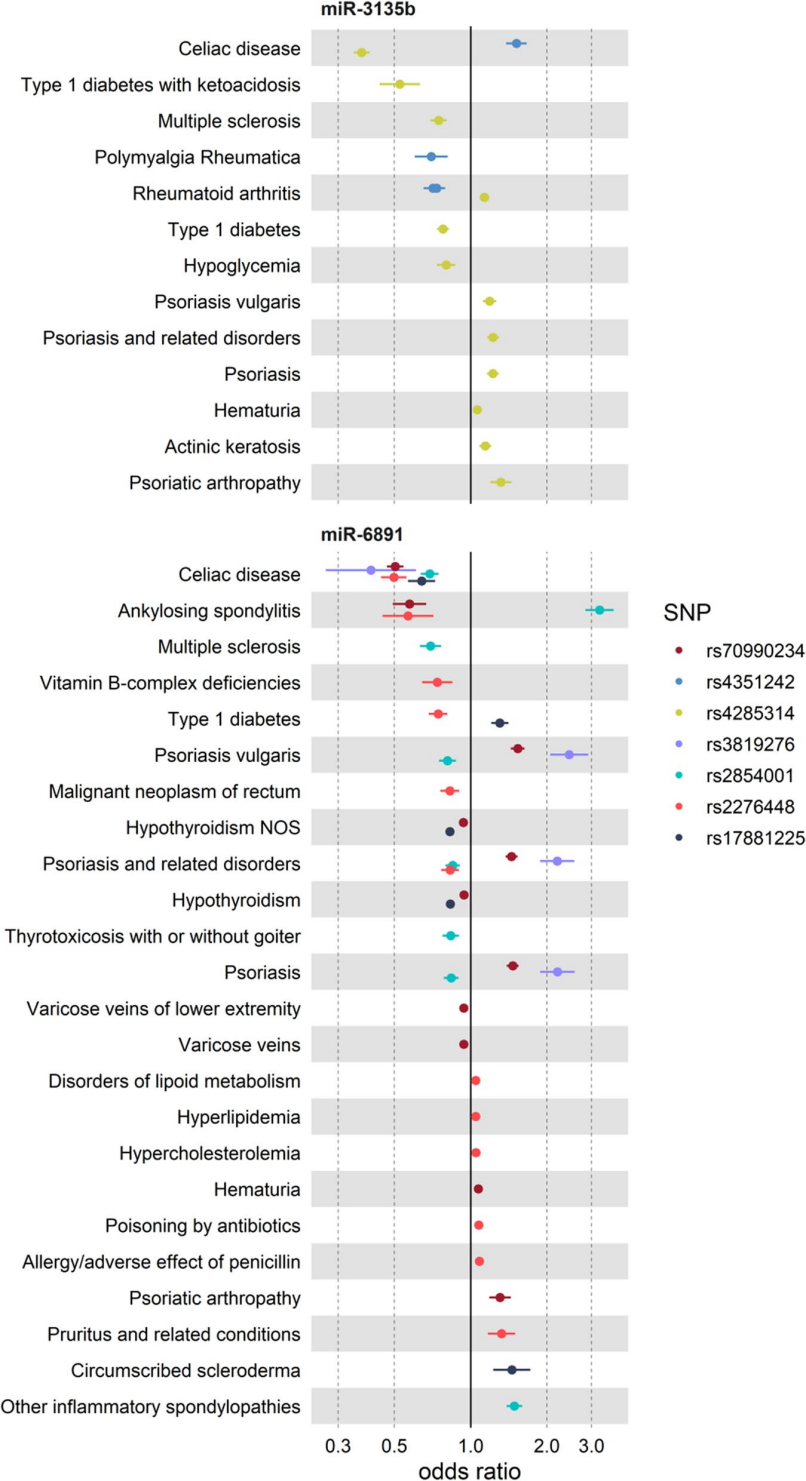
Forest-plots for FDR-significant associations of genetic variants located in the MHC region, including for miR-6891 and miR-3135b precursors. Dots represent odds ratio (OR) and 95%CI of the association between the variant (shown by different colour) and clinical conditions. The chromosomal position and corresponding effect alleles are presented in Additional file 1: Table S2

Fifty-seven associations (46.7%) identified in our analysis were for seven variants in the major histocompatibility complex (MHC) region (Fig. 2, Additional file 1: Table S2). Each variant in this region was associated with multiple diagnoses in at least two disease groups (Additional file 1: Fig. S2), the majority were immune-related disorders (Fig. 3, Additional file 1: Table S2). Examples are between rs2276448 and celiac disease (OR 0.49, *P* = 1.7 × 10^–32^), ankylosing spondylitis (OR 0.57, *P* = 1.2 × 10^–6^), and allergy to antibiotics (OR 1.08, *P* = 1.2 × 10^–7^).

**Fig. 4:**
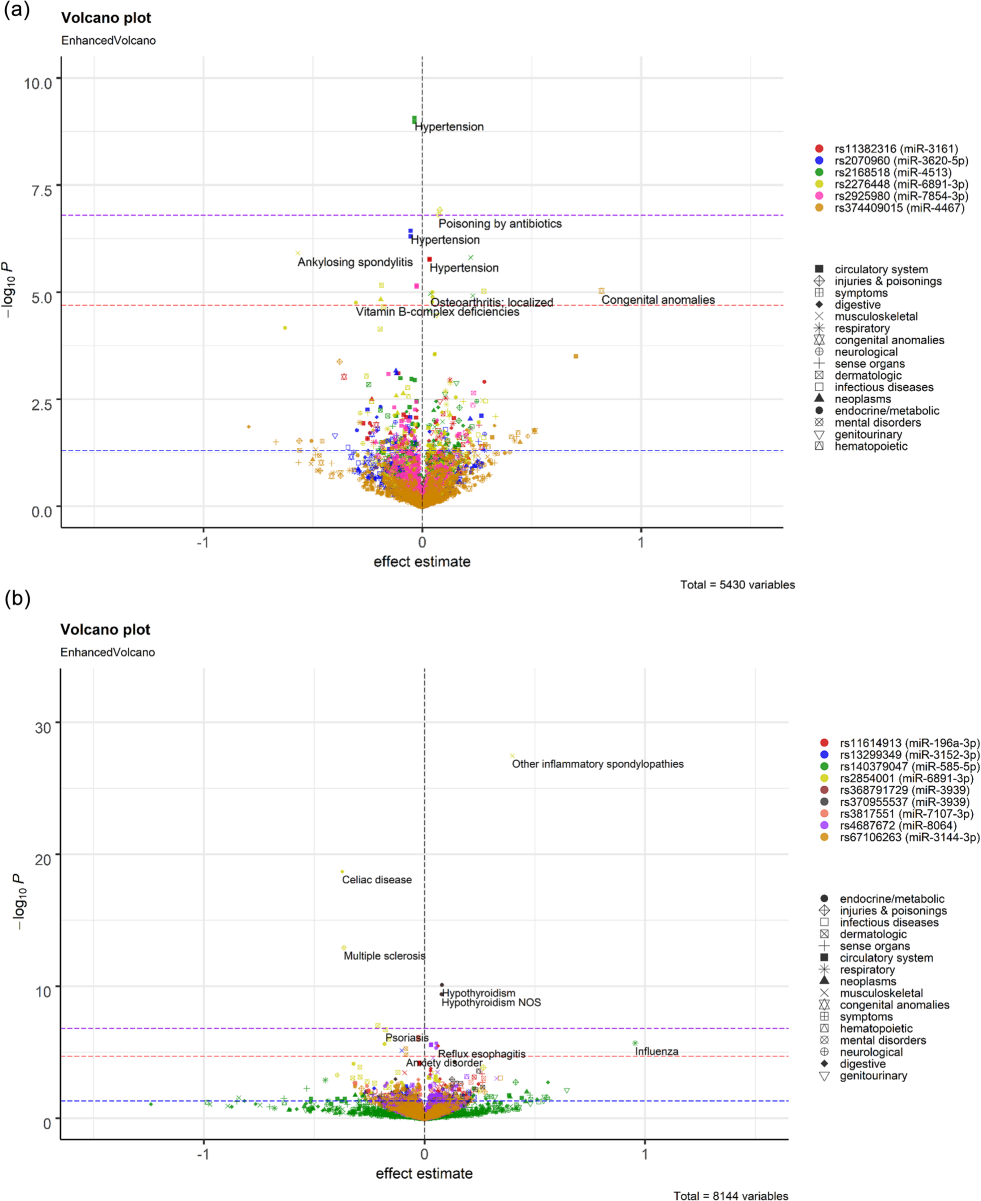

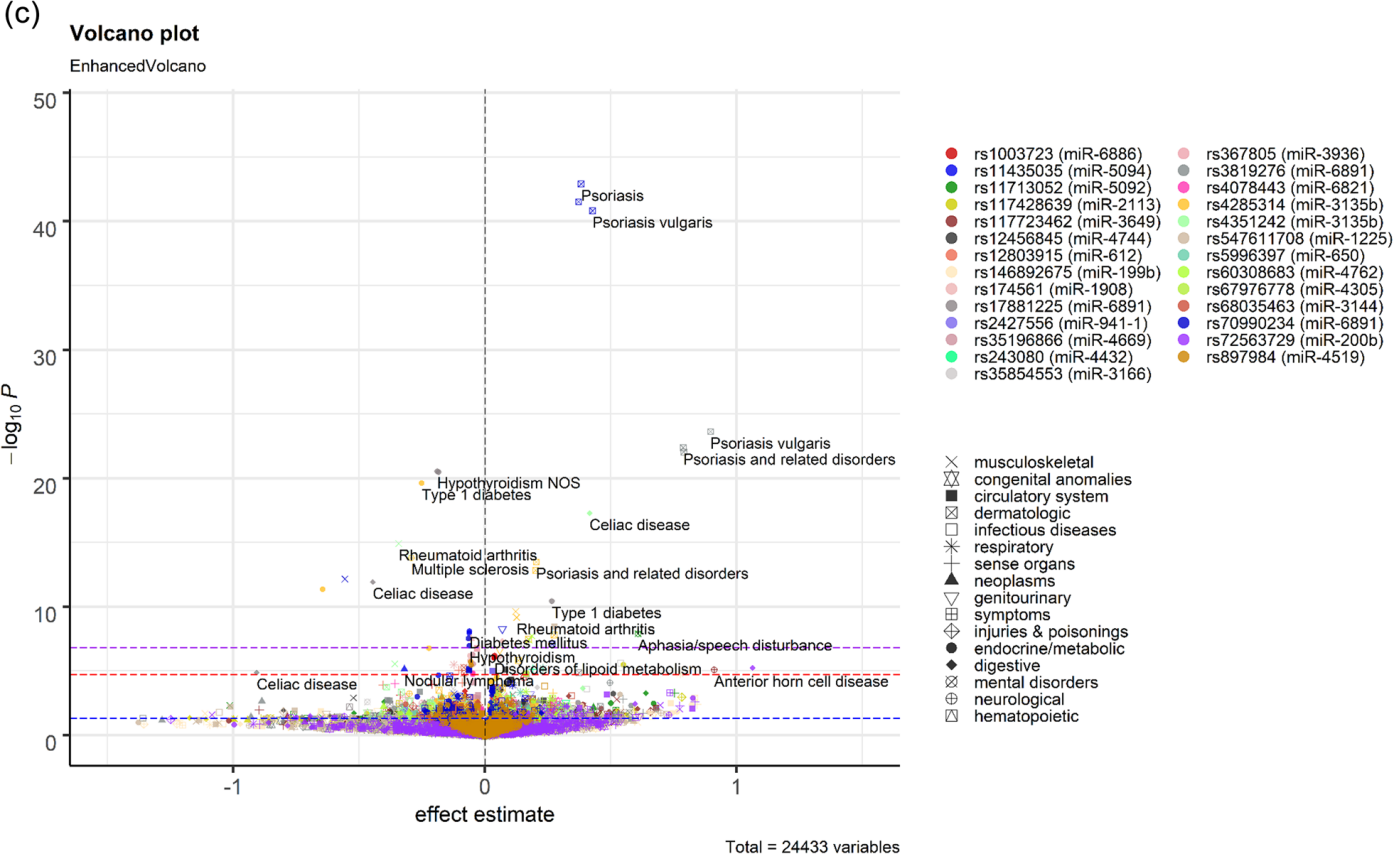
Enhanced volcano plots for PheWAS results of SNPs in seed (**a**), mature (**b**), or precursor genes (**c**) of miRNAs. Plots were only created for variants with at least one FDR-significant finding and shown for those with *P* > 1 × 10^–50^. Full results with allele information are provided in Additional file 1: Table S2. The X-axis denotes effect estimates (log odds ratio) for corresponding effect alleles. *Y* axis indicates − log10 of the association *p* values between each variant and clinical condition. Different colours of the dots represent different variants. Different shapes show different disease groups. Thresholds of significance are indicated by dashed blue (nominal), red (FDR < 0.05), and purple (Bonferroni or *P* < 0.05/(346 × 905)) lines

Outside the MHC region, 12 associations were with variants in seed, 15 in mature, and 38 in the precursor regions of miRNAs (Fig. 4). Several variants in the seed region were associated with hypertension, namely rs2168518 in miR-4513 (OR 0.96, *P* = 8.5 × 10^–10^), rs2070960 in miR-3620-5p (OR 0.95, *P* = 3.7 × 10^–7^), rs2925980 in miR-7854-3p (OR 0.97, *P* = 7.0 × 10^–6^), and rs11382316 in miR-316 (OR 1.03, *P* = 1.7 × 10^–6^). The rs2168518 polymorphism in miR-4513 was additionally associated with musculoskeletal disorders, including osteoarthritis (OR 1.04, *P* = 1.1 × 10^–5^). To note, the associations between rs2168518 and hypertension were significant after more stringent corrections for multiple testing using Bonferroni method (*P* < 0.05/(346 × 905) (Fig. 4a, Additional file 1: Table S2).

In the mature region, two variants in miR-3939 were associated with hypothyroidism, namely rs368791729 (OR 1.08, *P* = 8.1 × 10^–11^) and rs370955537 (OR 1.08, *P* = 8.1 × 10^–11^). Multiple variants were associated with hypertension, namely rs3817551 in miR-7107-3p (OR 0.97, *P* = 1.1 × 10^–6^) and rs4687672 in miR-8064 (OR 1.03, *P* = 2.9 × 10^–6^). Additionally, rs4687672 was also associated with type 2 diabetes (OR 1.05, *P* = 4.8 × 10^–6^). Rs11614913 in the mature region of miR-196a was previously associated with survival in non-small cell lung cancers [41], decreased risk of breast cancer [42], and type 2 diabetes [43]. Here, we additionally showed rs11614913 being associated with reflux esophagitis (OR 1.06, *P* = 3.3 × 10^–6^) (Fig. 4b, Additional file 1: Table S2). Of the 38 associations for 22 variants in precursor genes of 22 miRNAs, the strongest association was between rs547611708 in the precursor of miR-1225 and atrial fibrillation and flutter (OR 1.32, *P* = 3.6 × 10^–9^) (Fig. 4c, Additional file 1: Table S2).

### Target gene and enrichment analysis

As genetic variants in the seed region of miRNAs are likely to affect the miRNA-target mRNA binding, putative target genes for miRNAs with findings in their seed region were investigated by leveraging the genome-wide association studies (GWAS) hits for relevant traits. To this end, we used the genetic variants located in the target genes of miRNAs and tested their association with corresponding traits.

Among associations in the seed region of six miRNAs (miR-3161, miR-3620-5p, miR-4467, miR-4513, miR-6891-3p, miR-7854-3p) (Additional file 1: Table S2), genome-wide significant hits from previous GWAS with publicly available summary statistics on blood pressure [44], cholesterol levels [45], and osteoarthritis [46] were used in combination with predicted and validated target genes from TargetScan v7.2 [36] and miRTarBase [37]. Target genes that might mediate the association between miR-3620-5p, miR-3161, and miR-4513 with hypertension and between miR-6891-3p and cholesterol levels were identified (Additional file 1: Table S3). We also found the proportion of target genes associated with blood pressure is higher than expected for miR-3620-3p (*P* = 0.01).

### miRNA expression quantitative trait loci look-up

We used summary statistics of miRNA expression quantitative trait loci (miR-eQTLs) [38, 39] to check whether the studied variants are associated with the blood or plasma levels of their corresponding miRNAs. The genetic variants in the precursor region of miRNAs were mapped into both -3p and -5p forms of mature miRNAs. Out of 346 variants tested in current analysis, 33 variants were nominally significant of which 13 variants were found to be miR-eQTLs of corresponding miRNAs after Bonferroni correction (*P* < 0.05/346) (Additional file 1: Table S4).

### Colocalisation and Mendelian randomisation analysis

We conducted colocalisation and Mendelian randomisation (MR) analysis for findings with significant miR-eQTLs and the clinical conditions where large GWAS summary statistics are available. We used summary statistics for type 2 diabetes from DIAGRAM [47], type 1 diabetes from Forgetta et al. [48], and lipid traits from Global Lipid Genetics Consortium (GLGC) (total cholesterol (TC), triglyceride (TG), low-density lipoprotein cholesterol (LDL-C), high-density lipoprotein cholesterol (HDL-C)) [45] to conduct colocalisation for miR-6891-3p and miR-6821-5p (Additional file 1: Table S5). We found evidence for colocalisation between miR-6891-3p and total cholesterol (PP H4 > 0.9). Suggestive evidence was also found between miR-6891-3p and triglycerides (PP H4 > 0.6) and HDL-C (PP H4 > 0.6). The same shared causal variant was identified for association between miR-6891-3p and TC and HDL-C (rs2596501), but not for TG (rs3130614) (Additional file 1: Table S5, Fig. S3). Further, our single-instrument MR analysis suggested protective effect of miR-6891-3p on the risk of hyperlipidaemia (OR 0.69, *P* = 1.0 × 10^–5^) using the UK Biobank as the outcome dataset. This finding was replicated using the largest GWAS from GLGC as the outcome dataset [49], supporting the lowering effect of miR-6891-3p on TC, TG, LDL-C, and non-HDL-C (Table 3).

**Table 3:** Mendelian randomisation analysis

SNP	EA	Exposure	SNP-miRNA association			SNP-outcome association				miRNA-outcome association		
			Beta	SE	*P*	Outcome	Beta	SE	*P*	Beta	SE	*P*
*Outcome data from the UK Biobank*												
rs2276448	T	miR-6891-3p	0.13	0.03	2.39 × 10^–6^	Hyperlipidemia	− 0.046	0.011	1.01 × 10^–5^	− 0.37	0.08	1.01 × 10^–5^
*Outcome data from GLGC*												
rs2276448	T	miR-6891-3p	0.13	0.03	2.39 × 10^–6^	HDL-C	− 0.003	0.002	1.42 × 10^–1^	− 0.02	0.02	1.42 × 10^–1^
						TG	− 0.010	0.002	6.46 × 10^–7^	− 0.08	0.02	6.46 × 10^–7^
						TC	− 0.019	0.002	4.79 × 10^–24^	− 0.15	0.01	4.79 × 10^–24^
						LDL-C	− 0.017	0.002	2.87 × 10^–19^	− 0.14	0.02	2.87 × 10^–19^
						Non-HDL-C	− 0.018	0.002	4.51 × 10^–14^	− 0.14	0.02	4.51 × 10^–14^

*EA* effect allele, *SE* standard error, *GLGC* global lipid genetics consortium, *TC* total cholesterol, *TG* triglyceride, *LDL-C* low-density lipoprotein cholesterol, *HDL-C* high-density lipoprotein cholesterol, *MR* analysis was conducted using Wald ratio method

## Discussion

This study presented an agnostic investigation of 346 common genetic variants residing in miRNA-related sequences against a wide range of clinical diagnoses. We highlighted the top findings between variants in the MHC region with a range of diseases, the majority belonging to immune-related disorders. We demonstrated the value of phenome-wide analysis in unravelling the pleiotropy of miRNAs by studying a wide range of conditions beyond what has been previously investigated. For example, rs2168518 in the seed region of miR-4513 was known mainly for its association with the risk of cardiometabolic phenotypes, including fasting glucose, LDL-C, total cholesterol, and risk of coronary artery disease [50]. In the current analysis, rs2168518 was also associated with osteoarthritis and intervertebral disc disorders. As another example, rs11614913, located in the mature region of miR-196a-3p, which was also known to be related to cardiometabolic traits [43, 51] and the risk of several cancers [41, 42], was associated with reflux esophagitis in this analysis. Several findings belong to miRNAs that were more recently discovered in humans, such as miR-5094, miR-6821, miR-7107-3p, and miR-8064 and, thus, have not been extensively studied in the literature.

Nearly half of the associations identified in our analysis were for variants in the MHC region. We highlighted potential causal associations between miR-6891-3p and hyperlipidaemia through colocalisation and MR analysis. miR-6891-3p is located in the MHC region, known as the most-dense region in the human genome that contains a rich density of genes, is highly polymorphic, and has complex linkage disequilibrium (LD) structure that differs across populations [52]. The proteins encoded by human leukocyte antigen (*HLA*) genes in this region are essential in immune response, contributing to the pleiotropic role of this region in infectious, inflammatory, autoimmune diseases [53], and neurological disorders [54]. The genetic variants in the MHC region were previously shown to contribute substantially to genome-wide association analysis across an extended range of phenotypes in the UK Biobank [55]. Current findings validate our approach by replicating the known pleiotropic nature of this region [56, 57]. Three variants in miR-6891-3p with significant PheWAS findings also affect the expression of *HLA-B* [58], the host gene where miR-6891-3p resides in its intron [59], in addition to many other nearby genes. The extensive LD structure in the MHC region makes it challenging to assign causal variants since multiple variants in high LD could have nearly equivalent statistical associations [52, 60]. For a particular disease risk, there could be interactive effects of different alleles within the same locus or across different loci (epistasis) [61, 62].

Our colocalisation and MR analysis indicated the presence of shared causal variant and potential aetiological effect of miR-6891-3p on the risk of hyperlipidaemia. While one of the assumptions for colocalisation is that the region is densely genotyped and that the true causal variant is among these genotyped variants [40], the complex LD structure in the MHC region and the absence of substantial proportion of well-imputed variants in the tested region could have led to undetected shared causal variants between miR-6891-3p and other conditions reported here. A previous study showed miR-6891-3p targeting *TLR4*, thereby inhibiting inflammatory response in osteoarthritis [63]. For its counterpart, the target site of miR-6891-5p in *IGHA1* and *IGHA2* was identified, supporting the role of miR-6891-5p in IgA deficiency [64]. In vitro experiments showed that rs4351242 reduced the level of miR-3135b and was associated with age-related macular degeneration [8], while rs4285314 was associated with the risk of rheumatoid arthritis in the Chinese population [65]. We highlighted putative target genes relevant to the diseases for the associations identified in seed region, in line with the concept that target genes for miRNAs tend to be clustered according to their function [26]. These candidate target genes might be of interest for further studies to dissect underlying mechanisms. Our findings also indicate the value of using population-based genetic and clinical data to conduct a hypothesis-generating approach and complement those from experimental studies.

This analysis has several strengths by taking advantage of PheWAS as a powerful method to discover novel associations beyond what is known in the literature and reveal the pleiotropy of a genetic variant and miRNAs [66]. First, the UK Biobank has been deeply phenotyped and is genotyped using similar genotyping arrays, ensuring uniformity in the data and making it less likely to find spurious associations. Nevertheless, for some traits such as cancers, the UK Biobank might not have sufficient number of cases. To maximise power in our analysis, we utilised the full set of UK Biobank participants with available genotype and clinical diagnoses data, excluding related individuals and those who have withdrawn their consent. Although there is a higher proportion of females in our study (54%), we adjusted for sex, among other covariates in our analysis, which should have minimised the effect of confounding due to sex. Second, the use of ICD classification could minimise the misclassification of cases and controls compared to a standard self-reported based assignment. Third, clinical data could best represent the clinical importance of genotype to phenotype associations. A genetic variant could be associated with phenotype, but that does not always imply its clinical relevance.

This study has several limitations. First, the current investigation is limited to clinical conditions that belong to binary traits. PheWAS could also be implemented on different outcomes, such as laboratory measurement or imaging data [67]. Second, the current analysis is limited to miRNAs with common variants. Among 346 variants tested, only a small proportion was found to affect corresponding mature miRNA levels. The remaining variants might affect the functionality rather than the level of miRNAs. As we are focusing on common variants, this observation may also imply that miRNAs have a tight constraint on their expression such that common variants do not show many effects. Most candidate variants are rare and, thus, are not available in our current dataset. This highlights the need to analyse the effect of rare variants on miRNA expression and their consequences on human diseases, such as by using whole-genome sequencing data.

Third, it is important to ascertain whether the same genetic variant affects miRNA level in tissues that are relevant to the diseases. When tissue-specific miR-eQTLs become available, we could check whether the same candidate variant affects the expression of miR-6891-3p in adipose tissue and conduct colocalisation and MR analysis using this data. An experimental study could also test whether the presence of certain allele of the candidate variant influences the corresponding miRNA abundance through transfection experiments [8, 68]. Our study could help in prioritising candidate miRNAs for in-depth candidate-based experiments in relevant cell or tissue type.

Fourth, since our study focused on participants of European ancestries, it is essential to replicate our findings in different ancestral groups. Incorporating diverse ancestries could generate more transferrable findings for a wider population. Finally, despite using the largest GWAS on miRNA to date, we were not able to study all candidates and larger sample size might be needed to identify strong instruments for miRNAs. Therefore, we cannot rule out a possibility of undetected causal relationship between miRNAs and the diseases reported.

## Conclusions

Our study offers an opportunity to investigate the effect of variants in miRNAs and discover their clinical importance using population-level data. We identified a potential causal effect of miR-6891-3p on the risk of hyperlipidaemia, as consistently supported by PheWAS, colocalisation, and MR analysis, highlighting the value of using large population genetic and clinical data to study the causal role of miRNAs in human diseases.

### Supplementary Information

**Additional file 1. Table S1:** The list of 346 genetic variants tested in PheWAS. **Table S2:** 122 FDR-significant hits in PheWAS. **Table S3:** Candidate target genes whose interaction might be implicated by the presence of SNPs in seed region of miRNAs. **Table S4:** Genetic variants in miRNAs that affect plasma levels of corresponding mature miRNAs (P<0.05). **Table S5:** Results of colocalisation analysis. **Fig. S1:** Selection of study participants. **Fig. S2:** Each tested variant in MHC is associated with multiple disease groups. **Fig. S3:** Regional plot for colocalisation analysis between miR-6891-3p and total cholesterol (**a**), triglycerides (**b**), and high-density lipoprotein cholesterol (HDL-C). Variants were filtered based on imputation quality (Rsq>0.7). The most likely shared causal variant is labelled.

## Data Availability

Access to the individual-level data in UK Biobank is dependent on an application directly to the UK Biobank through its standard application process (https://www.ukbiobank.ac.uk/).

## Abbreviations

DIAGRAM: Diabetes genetics replication and meta-analysis
eQTL: Expression quantitative trait loci
GLGC: Global lipids genetics consortium
GWAS: Genome-wide association study
HDL-C: High-density lipoprotein cholesterol
HES: Hospital episode statistics
ICD: International classification of disease
LD: Linkage disequilibrium
LDL-C: Low-density lipoprotein cholesterol
MHC: Major histocompatibility region
miRNA: MicroRNA
OR: Odds ratio
PheWAS: Phenome-wide association study
RNA: Ribonucleic acid
SE: Standard error
TC: Total cholesterol
TG: Triglycerides
