# Supplementary material for: Phenome-wide association study on miRNA-related sequence variants: the UK Biobank
Source: Hum Genomics. 2023 Nov 24;17:104. doi: 10.1186/s40246-023-00553-w (PMC10668386; doi:10.1186/s40246-023-00553-w)
Supplement: Supplementary file 1 — Additional file 1. Table S1: The list of 346 genetic variants tested in PheWAS. Table S2: 122 FDR-significant hits in PheWAS. Table S3: Candidate target genes whose interaction might be implicated by the presence of SNPs in seed region of miRNAs. Table S4: Genetic variants in miRNAs that affect plasma levels of corresponding mature miRNAs (P<0.05). Table S5: Results of colocalisation analysis. Fig. S1: Selection of study participants. Fig. S2: Each tested variant in MHC is associated with multiple disease groups. Fig. S3: Regional plot for colocalisation analysis between miR-6891-3p and total cholesterol (a), triglycerides (b), and high-density lipoprotein cholesterol (HDL-C). Variants were filtered based on imputation quality (Rsq>0.7). The most likely shared causal variant is labelled. [file 40246_2023_553_MOESM1_ESM.pdf]

Additional file

Phenome-wide association study on miRNA-related sequence variants: the UK Biobank

Rima Mustafa, MD PhD[1,2,3,4], Mohsen Ghanbari, MD PhD[5], Ville Karhunen, PhD[6,7], Marina Evangelou, PhD[8], Abbas Dehghan, MD PhD[1,2,9]*

Affiliations

[1]Department of Epidemiology and Biostatistics, Imperial College London, London, UK.

[2]UK Dementia Research Institute, Imperial College London, London, UK.

[3]Big Data Institute, Li Ka Shing Centre for Health Information and Discovery, University of Oxford, Oxford, UK.

[4]Nuffield Department of Population Health, University of Oxford, Oxford, UK.

[5]Department of Epidemiology, Erasmus MC, Rotterdam, The Netherlands.

[6]Research Unit of Mathematical Sciences, University of Oulu, Oulu, Finland.

[7]Research Unit of Population Health, University of Oulu, Oulu, Finland.

[8]Department of Mathematics, Imperial College London, London, UK.

[9]MRC Centre for Environment and Health, Imperial College London, London, UK.

Running title: Phenome-wide association study of genetic variants in microRNAs

Corresponding authors

Abbas Dehghan, MD PhD

Department of Epidemiology & Biostatistics

Imperial College London

St Mary's Campus, Norfolk Place, W2 1PG

London, United Kingdom

Email: a.dehghan@imperial.ac.uk

Table S1. The list of 346 genetic variants tested in PheWAS

chr	bp	rsid	EA	EAF	location	pre_id	mature_id
1	1102563	rs72563729	A	0.99	pre-miRNA	hsa-mir-200b	NA
1	1231507	rs619608	A	0.95	Seed	hsa-mir-6726	hsa-miR-6726-3p
1	12251808	rs653667	G	0.64	pre-miRNA	hsa-mir-4632	NA
1	12639018	rs2275874	T	0.46	pre-miRNA	hsa-mir-6730	NA
1	17604437	rs72646786	T	0.86	pre-miRNA	hsa-mir-3972	NA
1	20960230	rs45530340	T	0.80	pre-miRNA	hsa-mir-6084	NA
1	54519800	rs74085143	A	0.98	Seed	hsa-mir-4781	hsa-miR-4781-3p
1	55691384	rs17111728	C	0.92	pre-miRNA	hsa-mir-4422	NA
1	65523519	rs521188	G	0.97	pre-miRNA	hsa-mir-3671	NA
1	67094171	rs12402181	A	0.84	Seed	hsa-mir-3117	hsa-miR-3117-3p
1	98510847	rs74904371	T	0.98	Seed	hsa-mir-2682	hsa-miR-2682-3p
1	117102649	rs1414273	T	0.88	pre-miRNA	hsa-mir-548ac	NA
1	178646884	rs56088671	T	0.93	pre-miRNA	hsa-mir-4424	NA
1	206648147	rs1953090	G	0.74	pre-miRNA	hsa-mir-6769b	NA
1	224585958	rs7522956	C	0.79	pre-miRNA	hsa-mir-4742	NA
1	228284991	rs2070960	T	0.92	Seed	hsa-mir-3620	hsa-miR-3620-5p
1	233759918	rs701213	C	0.79	pre-miRNA	hsa-mir-4427	NA
1	233759939	rs701214	T	0.91	pre-miRNA	hsa-mir-4427	NA
1	234442257	rs877722	T	0.90	pre-miRNA	hsa-mir-4671	NA
2	6790537	rs10192411	G	0.58	pre-miRNA	hsa-mir-7515	NA
2	12877501	rs5829384	GA	0.37	pre-miRNA	hsa-mir-3125	NA
2	35696519	rs62143301	A	0.89	Mature	hsa-mir-548ad	hsa-miR-548ad-3p
2	47604856	rs114803590	C	0.99	pre-miRNA	hsa-mir-559	NA
2	47604866	rs58450758	T	0.92	pre-miRNA	hsa-mir-559	NA
2	52929680	rs6726779	C	0.65	pre-miRNA	hsa-mir-4431	NA
2	56210140	rs41291173	A	0.98	Mature	hsa-mir-217	hsa-miR-217-3p
2	56216090	rs41291179	T	0.95	pre-miRNA	hsa-mir-216a	NA
2	60614572	rs243080	A	0.55	pre-miRNA	hsa-mir-4432	NA
2	60614577	rs56239160	G	0.95	pre-miRNA	hsa-mir-4432	NA
2	64567916	rs12473206	G	0.72	Seed	hsa-mir-4433b	hsa-miR-4433b-3p
2	64567916	rs12473206	G	0.72	Seed	hsa-mir-4433a	hsa-miR-4433a-5p
2	103048780	rs62154973	T	0.92	Mature	hsa-mir-4772	hsa-miR-4772-5p
2	114478923	rs113469098	T	0.99	Mature	hsa-mir-4782	hsa-miR-4782-5p
2	134884700	rs6430498	A	0.72	pre-miRNA	hsa-mir-3679	NA
2	141344200	rs56148568	C	0.78	Mature	hsa-mir-7157	hsa-miR-7157-3p
2	176032376	rs79402775	A	0.98	Mature	hsa-mir-933	hsa-miR-933
2	180725568	rs146754630	C	0.97	pre-miRNA	hsa-mir-1258	NA
2	207647981	rs2241347	T	0.83	Mature	hsa-mir-3130-1	hsa-miR-3130-5p
2	207647981	rs2241347	T	0.83	Mature	hsa-mir-3130-2	hsa-miR-3130-5p
2	207647981	rs2241347	T	0.83	Mature	hsa-mir-3130-2	hsa-miR-3130-3p
2	207647981	rs2241347	T	0.83	Mature	hsa-mir-3130-1	hsa-miR-3130-3p
2	208031167	rs6717413	A	0.34	pre-miRNA	hsa-mir-7845	NA
2	208134114	rs116237969	T	0.98	pre-miRNA	hsa-mir-1302-4	NA
2	219206645	rs62182086	G	0.86	Seed	hsa-mir-6810	hsa-miR-6810-5p
2	220771223	rs4674470	T	0.22	pre-miRNA	hsa-mir-4268	NA
2	238419611	rs2292879	G	0.77	Mature	hsa-mir-6811	hsa-miR-6811-3p
2	240007544	rs12233076	T	0.69	pre-miRNA	hsa-mir-4441	NA
2	240882476	rs78832554	A	0.97	pre-miRNA	hsa-mir-4786	NA
2	241395500	rs71428439	G	0.87	pre-miRNA	hsa-mir-149	NA
2	241395503	rs2292832	C	0.29	pre-miRNA	hsa-mir-149	NA
3	20179097	rs6787734	T	0.90	pre-miRNA	hsa-mir-3135a	NA
3	20179130	rs142342924	A	0.93	pre-miRNA	hsa-mir-3135a	NA
3	44903434	rs2292181	C	0.95	pre-miRNA	hsa-mir-564	NA
3	48587408	rs35452137	C	0.89	Seed	hsa-mir-6823	hsa-miR-6823-3p
3	52880543	rs4687672	A	0.77	Mature	hsa-mir-8064	hsa-miR-8064
3	96078864	rs1514422	A	0.95	Mature	hsa-mir-8060	hsa-miR-8060
3	120768492	rs9877402	G	0.97	pre-miRNA	hsa-mir-5682	NA
3	124093220	rs78790512	A	0.83	pre-miRNA	hsa-mir-6083	NA
3	124451312	rs10934682	G	0.82	pre-miRNA	hsa-mir-544b	NA
3	124870376	rs11713052	G	0.97	pre-miRNA	hsa-mir-5092	NA
3	128990998	rs6771809	C	0.90	Seed	hsa-mir-6826	hsa-miR-6826-5p
3	151283691	rs9842591	A	0.53	pre-miRNA	hsa-mir-5186	NA
3	175087408	rs78831152	T	0.88	pre-miRNA	hsa-mir-4789	NA

3	189547735	rs75715827	C	0.94	pre-miRNA	hsa-mir-944	NA
3	197020780	rs3836429	GA	0.39	pre-miRNA	hsa-mir-4797	NA
4	1988184	rs201953811	ACC	0.79	pre-miRNA	hsa-mir-943	NA
4	1988188	rs3034718	CCT	0.79	pre-miRNA	hsa-mir-943	NA
4	1988193	rs1077020	C	0.78	pre-miRNA	hsa-mir-943	NA
4	5925054	rs28645567	A	0.97	pre-miRNA	hsa-mir-378d-1	NA
4	7461769	rs12512664	G	0.58	pre-miRNA	hsa-mir-4274	NA
4	36428048	rs6841938	A	0.92	Mature	hsa-mir-1255b-1	hsa-miR-1255b-5p
4	67142620	rs73239138	A	0.74	Mature	hsa-mir-1269a	hsa-miR-1269a
4	102251501	rs28664200	C	0.71	pre-miRNA	hsa-mir-1255a	NA
4	110409933	rs77639117	T	0.96	pre-miRNA	hsa-mir-576	NA
4	115577997	rs34115976	G	0.82	pre-miRNA	hsa-mir-577	NA
5	1708983	rs12523324	A	0.35	pre-miRNA	hsa-mir-4277	NA
5	9053945	rs257095	T	0.13	pre-miRNA	hsa-mir-4636	NA
5	54466544	rs10061133	G	0.91	Mature	hsa-mir-449b	hsa-miR-449b-5p
5	54468124	rs35770269	T	0.68	Seed	hsa-mir-449c	hsa-miR-449c-3p
5	57825920	rs10461441	G	0.91	Mature	hsa-mir-548ae-2	hsa-miR-548ae-5p
5	71465361	rs3112399	A	0.59	pre-miRNA	hsa-mir-4803	NA
5	72174432	rs266435	G	0.14	Seed	hsa-mir-4804	hsa-miR-4804-5p
5	82136024	rs79512808	G	0.98	pre-miRNA	hsa-mir-3977	NA
5	89312487	rs75102645	A	0.98	pre-miRNA	hsa-mir-3660	NA
5	115591460	rs77613551	CA	0.85	pre-miRNA	hsa-mir-12130	NA
5	131701279	rs367805	C	0.30	pre-miRNA	hsa-mir-3936	NA
5	143059433	rs2042253	C	0.76	pre-miRNA	hsa-mir-5197	NA
5	150901699	rs3734050	T	0.91	Seed	hsa-mir-6499	hsa-miR-6499-5p
5	153726769	rs13186787	G	0.98	pre-miRNA	hsa-mir-1294	NA
5	153975576	rs936581	A	0.83	pre-miRNA	hsa-mir-3141	NA
5	154065348	rs77055126	C	0.98	pre-miRNA	hsa-mir-1303	NA
5	154065383	rs75538180	T	0.64	pre-miRNA	hsa-mir-1303	NA
5	154209024	rs702742	G	0.91	pre-miRNA	hsa-mir-378h	NA
5	159901483	rs747499864	CCT	0.98	pre-miRNA	hsa-mir-3142	NA
5	159912418	rs2910164	G	0.23	Seed	hsa-mir-146a	hsa-miR-146a-3p
5	168690612	rs62376934	G	0.36	pre-miRNA	hsa-mir-585	NA
5	168690635	rs62376935	T	0.94	Seed	hsa-mir-585	hsa-miR-585-3p

5	168690664	rs140379047	G	0.99	Mature	hsa-mir-585	hsa-miR-585-5p
5	174178774	rs7709117	A	0.40	pre-miRNA	hsa-mir-4634	NA
5	179225324	rs2291418	A	0.96	Mature	hsa-mir-1229	hsa-miR-1229-5p
5	180649616	rs146528803	A	0.98	Mature	hsa-mir-4638	hsa-miR-4638-5p
6	6169656	rs7769202	C	0.66	pre-miRNA	hsa-mir-7853	NA
6	10439968	rs9295535	C	0.79	Mature	hsa-mir-5689	hsa-miR-5689
6	18572056	rs12197631	G	0.95	pre-miRNA	hsa-mir-548a-1	NA
6	31323012	rs2854001	A	0.77	Mature	hsa-mir-6891	hsa-miR-6891-3p
6	31323020	rs2276448	C	0.86	Seed	hsa-mir-6891	hsa-miR-6891-3p
6	31323027	rs3819276	T	0.99	pre-miRNA	hsa-mir-6891	NA
6	31323039	rs70990234	TGA<br>AGG<br>GCT<br>CCA	0.65	pre-miRNA	hsa-mir-6891	NA
6	31323065	rs17881225	C	0.90	pre-miRNA	hsa-mir-6891	NA
6	32717702	rs4285314	A	0.54	pre-miRNA	hsa-mir-3135b	NA
6	32717722	rs4351242	T	0.92	pre-miRNA	hsa-mir-3135b	NA
6	44403438	rs67182313	G	0.85	pre-miRNA	hsa-mir-4642	NA
6	76138146	rs5877455	CAG	0.33	pre-miRNA	hsa-mir-4463	NA
6	98472445	rs117428639	T	0.98	pre-miRNA	hsa-mir-2113	NA
6	120336327	rs68035463	A	0.76	pre-miRNA	hsa-mir-3144	NA
6	120336384	rs67106263	A	0.76	Mature	hsa-mir-3144	hsa-miR-3144-3p
6	167411321	rs368791729	G	0.62	Mature	hsa-mir-3939	hsa-miR-3939
6	167411322	rs370955537	C	0.62	Mature	hsa-mir-3939	hsa-miR-3939
7	2297212	rs62442513	T	0.83	pre-miRNA	hsa-mir-6836	NA
7	7106600	rs6943868	A	0.97	pre-miRNA	hsa-mir-3683	NA
7	7106636	rs6977967	G	0.86	pre-miRNA	hsa-mir-3683	NA
7	64139463	rs7804972	A	0.60	Mature	hsa-mir-6839	hsa-miR-6839-5p
7	64139524	rs35559940	A	0.07	pre-miRNA	hsa-mir-6839	NA
7	100802786	rs11983381	G	0.84	pre-miRNA	hsa-mir-4653	NA
7	102106201	rs3823658	A	0.87	Seed	hsa-mir-5090	hsa-miR-5090
7	102111925	rs374409015	T	0.96	Seed	hsa-mir-4467	hsa-miR-4467
7	102111936	rs60871950	A	0.55	Mature	hsa-mir-4467	hsa-miR-4467
7	129410227	rs76481776	T	0.91	pre-miRNA	hsa-mir-182	NA
7	142157358	rs361399	G	0.29	pre-miRNA	hsa-mir-11400	NA

7	142157371	rs361398	G	0.29	pre-miRNA	hsa-mir-11400	NA
7	142157389	rs361397	A	0.71	Mature	hsa-mir-11400	hsa-miR-11400
7	143079806	rs6464546	A	0.40	pre-miRNA	hsa-mir-6892	NA
7	158325503	rs4909237	T	0.85	pre-miRNA	hsa-mir-595	NA
7	158384368	rs80128580	A	0.96	pre-miRNA	hsa-mir-5707	NA
8	1765425	rs61388742	C	0.89	Mature	hsa-mir-596	hsa-miR-596
8	9599276	rs79397096	A	0.99	pre-miRNA	hsa-mir-597	NA
8	24811322	rs76347846	G	0.92	Mature	hsa-mir-6841	hsa-miR-6841-3p
8	26906402	rs73235381	T	0.98	Mature	hsa-mir-548h-4	hsa-miR-548h-3p
8	26906437	rs184537764	G	0.97	pre-miRNA	hsa-mir-548h-4	NA
8	27559214	rs66683138	A	0.78	Seed	hsa-mir-3622b	hsa-miR-3622b-3p
8	27559214	rs66683138	A	0.78	Seed	hsa-mir-3622a	hsa-miR-3622a-5p
8	94041998	rs404337	A	0.18	Mature	hsa-mir-8084	hsa-miR-8084
8	99405919	rs78979347	A	0.99	pre-miRNA	hsa-mir-9903	NA
8	103137663	rs12549434	C	0.96	pre-miRNA	hsa-mir-5680	NA
8	103137693	rs487571	C	0.39	pre-miRNA	hsa-mir-5680	NA
8	113655752	rs10505168	C	0.71	pre-miRNA	hsa-mir-2053	NA
8	120337452	rs764337957	T	0.32	pre-miRNA	hsa-mir-548az	NA
8	129021179	rs2114358	A	0.42	pre-miRNA	hsa-mir-1206	NA
8	130496365	rs6997249	A	0.68	pre-miRNA	hsa-mir-3686	NA
8	143257760	rs28655823	C	0.90	Seed	hsa-mir-4472-1	hsa-miR-4472
8	145625537	rs554633373	G	0.98	pre-miRNA	hsa-mir-1234	NA
9	18573360	rs13299349	A	0.68	Mature	hsa-mir-3152	hsa-miR-3152-3p
9	20502274	rs74428911	T	0.99	pre-miRNA	hsa-mir-4474	NA
9	91360776	rs540869781	A	0.99	pre-miRNA	hsa-mir-4289	NA
9	94398581	rs67339585	C	0.89	pre-miRNA	hsa-mir-3910-2	NA
9	94398581	rs67339585	C	0.89	pre-miRNA	hsa-mir-3910-1	NA
9	97572244	rs356125	A	0.95	pre-miRNA	hsa-mir-2278	NA
9	126247833	rs58605477	G	0.78	pre-miRNA	hsa-mir-7150	NA
9	126247896	rs140297188	ACC<br>GTG<br>TGT<br>GTG<br>TGT<br>GC<br>GC	0.79	pre-miRNA	hsa-mir-7150	NA
9	131007001	rs146892675	T	0.99	pre-miRNA	hsa-mir-199b	NA
9	135821099	rs56195815	T	0.77	pre-miRNA	hsa-mir-548aw	NA
9	137271318	rs35196866	A	0.24	pre-miRNA	hsa-mir-4669	NA
9	137741480	rs113454901	A	0.19	pre-miRNA	hsa-mir-3689d-1	NA
9	137741993	rs34933499	A	0.97	pre-miRNA	hsa-mir-3689b	NA
9	137742597	rs72502717	G	0.85	pre-miRNA	hsa-mir-3689f	NA
9	139641414	rs138511229	G	0.98	pre-miRNA	hsa-mir-6722	NA
9	139781193	rs116932476	A	0.99	pre-miRNA	hsa-mir-4479	NA
10	12172775	rs7070684	A	0.55	pre-miRNA	hsa-mir-548ak	NA
10	12695177	rs7896283	G	0.53	pre-miRNA	hsa-mir-4481	NA
10	14425204	rs12780876	A	0.71	pre-miRNA	hsa-mir-4293	NA
10	14478618	rs11259096	C	0.95	pre-miRNA	hsa-mir-1265	NA
10	24564653	rs11014002	T	0.94	pre-miRNA	hsa-mir-603	NA
10	28578229	rs11436116	CA	0.91	pre-miRNA	hsa-mir-8086	NA
10	29833998	rs2368393	G	0.74	pre-miRNA	hsa-mir-604	NA
10	29834003	rs2368392	A	0.74	pre-miRNA	hsa-mir-604	NA
10	29891260	rs12416605	T	0.73	Seed	hsa-mir-938	hsa-miR-938
10	53059406	rs2043556	C	0.80	pre-miRNA	hsa-mir-605	NA
10	90823139	rs72810954	A	0.98	Mature	hsa-mir-4679-2	hsa-miR-4679
10	90823139	rs72810954	A	0.98	Mature	hsa-mir-4679-1	hsa-miR-4679
10	102734778	rs4919510	G	0.80	Mature	hsa-mir-608	hsa-miR-608
10	105154089	rs7911488	G	0.68	pre-miRNA	hsa-mir-1307	NA
10	106028154	rs45596840	A	0.69	Seed	hsa-mir-4482	hsa-miR-4482-5p
10	106028157	rs641071	T	0.64	pre-miRNA	hsa-mir-4482	NA
10	115933905	rs17091403	T	0.90	pre-miRNA	hsa-mir-2110	NA
11	1277894	rs56310773	T	0.97	Mature	hsa-mir-6744	hsa-miR-6744-3p
11	1880730	rs75966923	A	0.95	pre-miRNA	hsa-mir-4298	NA
11	10529822	rs7350542	G	0.85	pre-miRNA	hsa-mir-4485	NA
11	11804698	rs112932356	A	0.85	pre-miRNA	hsa-mir-8070	NA
11	11804757	rs138552125	T	0.97	Seed	hsa-mir-8070	hsa-miR-8070
11	19781594	rs77717467	A	0.99	pre-miRNA	hsa-mir-4694	NA
11	34963416	rs2986407	C	0.20	Mature	hsa-mir-1343	hsa-miR-1343-5p
11	34963459	rs11032942	C	0.89	pre-miRNA	hsa-mir-1343	NA
11	48118347	rs11382316	TA	0.21	Seed	hsa-mir-3161	hsa-miR-3161
11	56511354	rs67042258	A	0.74	pre-miRNA	hsa-mir-6128	NA

11	61582708	rs174561	C	0.69	pre-miRNA	hsa-mir-1908	NA
11	64646034	rs12801172	C	0.94	pre-miRNA	hsa-mir-10392	NA
11	65211940	rs550894	A	0.89	pre-miRNA	hsa-mir-612	NA
11	65211979	rs12803915	A	0.78	pre-miRNA	hsa-mir-612	NA
11	74110353	rs515924	G	0.85	Seed	hsa-mir-548al	hsa-miR-548al
11	79133220	rs11237828	C	0.81	Mature	hsa-mir-5579	hsa-miR-5579-3p
11	87909673	rs35854553	A	0.91	pre-miRNA	hsa-mir-3166	NA
11	94199710	rs11020790	T	0.99	pre-miRNA	hsa-mir-548l	NA
11	126858392	rs670637	G	0.77	pre-miRNA	hsa-mir-3167	NA
11	126858406	rs634171	T	0.77	pre-miRNA	hsa-mir-3167	NA
12	1769533	rs117723462	G	0.98	pre-miRNA	hsa-mir-3649	NA
12	47581629	rs832733	C	0.32	pre-miRNA	hsa-mir-4698	NA
12	47758032	rs215383	G	0.15	pre-miRNA	hsa-mir-4494	NA
12	54385599	rs11614913	T	0.58	Mature	hsa-mir-196a-2	hsa-miR-196a-3p
12	66417482	rs11176006	A	0.72	pre-miRNA	hsa-mir-6074	NA
12	66417493	rs10878362	A	0.69	pre-miRNA	hsa-mir-6074	NA
12	81329536	rs2682818	C	0.14	pre-miRNA	hsa-mir-618	NA
12	94955585	rs12314280	C	0.91	Mature	hsa-mir-5700	hsa-miR-5700
12	94955603	rs17022749	T	0.95	pre-miRNA	hsa-mir-5700	NA
12	94955607	rs75258105	T	0.92	pre-miRNA	hsa-mir-5700	NA
12	94965058	rs1290910	C	0.33	pre-miRNA	hsa-mir-7844	NA
12	95228286	rs2289030	C	0.94	pre-miRNA	hsa-mir-492	NA
12	104324266	rs17797090	A	0.91	pre-miRNA	hsa-mir-3652	NA
12	104985443	rs61938575	A	0.72	Mature	hsa-mir-3922	hsa-miR-3922-5p
12	116866114	rs11068023	C	0.61	Seed	hsa-mir-4472-2	hsa-miR-4472
12	120151493	rs7311975	C	0.96	Seed	hsa-mir-1178	hsa-miR-1178-5p
12	120151501	rs74614893	A	0.97	pre-miRNA	hsa-mir-1178	NA
12	121161048	rs1055070	G	0.95	Mature	hsa-mir-4700	hsa-miR-4700-3p
12	121882098	rs3817551	G	0.61	Mature	hsa-mir-7107	hsa-miR-7107-3p
12	121882141	rs55671311	G	0.92	Mature	hsa-mir-7107	hsa-miR-7107-5p
12	123849347	rs370987290	AGC<br>G	0.94	pre-miRNA	hsa-mir-8072	NA
12	128778703	rs1683709	A	0.82	pre-miRNA	hsa-mir-3612	NA
12	133158632	rs3751304	T	0.29	Seed	hsa-mir-6763	hsa-miR-6763-3p
13	40238175	rs67976778	T	0.62	pre-miRNA	hsa-mir-4305	NA
13	40238256	rs138201016	T	0.98	Seed	hsa-mir-4305	hsa-miR-4305
13	55748673	rs1572687	C	0.45	pre-miRNA	hsa-mir-5007	NA
13	66792439	rs76595065	C	0.97	Mature	hsa-mir-4704	hsa-miR-4704-3p
13	78272233	rs557214778	TGC<br>C	0.79	pre-miRNA	hsa-mir-3665	NA
14	21491532	rs117650137	A	0.96	Seed	hsa-mir-6717	hsa-miR-6717-5p
14	23426182	rs2273626	A	0.47	Seed	hsa-mir-4707	hsa-miR-4707-3p
14	50433227	rs35650931	C	0.91	pre-miRNA	hsa-mir-6076	NA
14	55344901	rs28477407	T	0.88	pre-miRNA	hsa-mir-4308	NA
14	65511421	rs72728267	T	0.98	Seed	hsa-mir-4706	hsa-miR-4706
14	100774203	rs72631832	T	0.98	pre-miRNA	hsa-mir-345	NA
14	101280856	rs73347569	G	0.98	pre-miRNA	hsa-mir-2392	NA
14	101489703	rs111906529	C	0.98	pre-miRNA	hsa-mir-411	NA
14	101507727	rs12894467	T	0.61	pre-miRNA	hsa-mir-300	NA
14	101522556	rs56103835	C	0.80	pre-miRNA	hsa-mir-323b	NA
14	101531854	rs61992671	G	0.51	Mature	hsa-mir-412	hsa-miR-412-3p
14	101533093	rs58834075	T	0.97	pre-miRNA	hsa-mir-656	NA
14	103006047	rs12879262	C	0.88	pre-miRNA	hsa-mir-4309	NA
15	66011630	rs2060455	G	0.82	pre-miRNA	hsa-mir-4511	NA
15	66011662	rs370142994	CT	0.84	pre-miRNA	hsa-mir-4511	NA
15	70371777	rs78212770	G	0.99	Mature	hsa-mir-629	hsa-miR-629-5p
15	75081078	rs2168518	A	0.32	Seed	hsa-mir-4513	hsa-miR-4513
15	75645965	rs766523130	C	0.92	pre-miRNA	hsa-mir-631	NA
15	79502168	rs41280052	T	0.98	pre-miRNA	hsa-mir-184	NA
15	86368898	rs4414449	A	0.37	Mature	hsa-mir-548ap	hsa-miR-548ap-5p
15	86368922	rs76468441	T	0.96	pre-miRNA	hsa-mir-548ap	NA
15	86368959	rs4577031	T	0.37	pre-miRNA	hsa-mir-548ap	NA
15	90393949	rs11435035	CT	0.30	pre-miRNA	hsa-mir-5094	NA
15	93447631	rs1439619	G	0.54	pre-miRNA	hsa-mir-3175	NA
16	593277	rs8054514	G	0.85	pre-miRNA	hsa-mir-3176	NA
16	593362	rs141861277	A	0.99	pre-miRNA	hsa-mir-3176	NA
16	2140262	rs547611708	TC	0.99	pre-miRNA	hsa-mir-1225	NA
16	15248720	rs75000738	A	0.19	pre-miRNA	hsa-mir-3180-4	NA
16	30886643	rs897984	C	0.39	pre-miRNA	hsa-mir-4519	NA
16	56938244	rs12708966	A	0.99	Seed	hsa-mir-6863	hsa-miR-6863

16	70064261	rs57629257	T	0.90	pre-miRNA	hsa-mir-1972-2	NA
16	76902842	rs58353328	G	0.97	pre-miRNA	hsa-mir-4719	NA
16	76902847	rs7500280	C	0.37	pre-miRNA	hsa-mir-4719	NA
16	76902850	rs7499278	A	0.25	pre-miRNA	hsa-mir-4719	NA
16	81567554	rs2925980	G	0.67	Seed	hsa-mir-7854	hsa-miR-7854-3p
16	81644970	rs74469188	C	0.89	Mature	hsa-mir-6504	hsa-miR-6504-5p
16	82722537	rs16958290	C	0.99	pre-miRNA	hsa-mir-8058	NA
16	87887560	rs77250474	A	0.80	pre-miRNA	hsa-mir-11401	NA
16	88535341	rs56292801	A	0.73	pre-miRNA	hsa-mir-5189	NA
16	88535407	rs35613341	G	0.67	Mature	hsa-mir-5189	hsa-miR-5189-3p
17	925742	rs72812091	A	0.92	pre-miRNA	hsa-mir-3183	NA
17	925764	rs2663345	G	0.69	pre-miRNA	hsa-mir-3183	NA
17	2596180	rs111246591	A	0.97	pre-miRNA	hsa-mir-6776	NA
17	6558768	rs8078913	T	0.52	Mature	hsa-mir-4520-1	hsa-miR-4520-5p
17	6558768	rs8078913	T	0.52	Mature	hsa-mir-4520-2	hsa-miR-4520-5p
17	6558768	rs8078913	T	0.52	Mature	hsa-mir-4520-1	hsa-miR-4520-3p
17	8090294	rs76800617	G	0.98	pre-miRNA	hsa-mir-4521	NA
17	12820632	rs12451747	C	0.43	Mature	hsa-mir-1269b	hsa-miR-1269b
17	12820646	rs7210937	C	0.92	Seed	hsa-mir-1269b	hsa-miR-1269b
17	13446924	rs9913045	A	0.62	Mature	hsa-mir-548h-3	hsa-miR-548h-5p
17	15154966	rs66507245	A	0.49	Seed	hsa-mir-4731	hsa-miR-4731-3p
17	28444183	rs6505162	C	0.56	pre-miRNA	hsa-mir-423	NA
17	29421443	rs17885221	T	0.97	pre-miRNA	hsa-mir-4733	NA
17	37453674	rs620301	A	0.42	pre-miRNA	hsa-mir-548bc	NA
17	40646803	rs11651671	A	0.72	Mature	hsa-mir-548at	hsa-miR-548at-5p
17	41522213	rs7207008	A	0.52	pre-miRNA	hsa-mir-2117	NA
17	58120418	rs200376243	GC	0.95	pre-miRNA	hsa-mir-4737	NA
17	61021611	rs17759989	G	0.97	pre-miRNA	hsa-mir-633	NA
17	72744798	rs745666	C	0.56	pre-miRNA	hsa-mir-3615	NA
17	74094105	rs7208391	G	0.55	Mature	hsa-mir-6868	hsa-miR-6868-3p
17	77681036	rs73410309	C	0.83	Mature	hsa-mir-4739	hsa-miR-4739
17	78072676	rs111756476	T	0.98	pre-miRNA	hsa-mir-1268b	NA
17	79107017	rs559410963	C	0.92	pre-miRNA	hsa-mir-1250	NA
18	5840810	rs72855836	A	0.95	pre-miRNA	hsa-mir-3976	NA
18	20513374	rs7227168	T	0.88	Seed	hsa-mir-4741	hsa-miR-4741
18	33484792	rs41274312	A	0.98	pre-miRNA	hsa-mir-187	NA
18	46196971	rs78396863	C	0.98	pre-miRNA	hsa-mir-4743	NA
18	46196998	rs7235219	G	0.99	pre-miRNA	hsa-mir-4743	NA
18	46576058	rs12456845	C	0.95	pre-miRNA	hsa-mir-4744	NA
18	56118358	rs41292412	T	0.99	Seed	hsa-mir-122	hsa-miR-122-3p
19	804959	rs10422347	T	0.91	Mature	hsa-mir-4745	hsa-miR-4745-5p
19	2434941	rs62122269	T	0.82	pre-miRNA	hsa-mir-7108	NA
19	6389699	rs78293125	G	0.90	Mature	hsa-mir-6885	hsa-miR-6885-5p
19	6416443	rs146253337	GT	0.95	pre-miRNA	hsa-mir-3940	NA
19	8454236	rs72996752	G	0.70	Mature	hsa-mir-4999	hsa-miR-4999-5p
19	11224181	rs1003723	T	0.54	pre-miRNA	hsa-mir-6886	NA
19	13031210	rs2967897	C	0.37	pre-miRNA	hsa-mir-5695	NA
19	13947292	rs895819	C	0.66	pre-miRNA	hsa-mir-27a	NA
19	13947296	rs11671784	A	0.99	pre-miRNA	hsa-mir-27a	NA
19	15290125	rs56061231	A	0.28	pre-miRNA	hsa-mir-6795	NA
19	18392894	rs7247237	T	0.73	pre-miRNA	hsa-mir-3188	NA
19	18392913	rs7247767	G	0.73	pre-miRNA	hsa-mir-3188	NA
19	35613623	rs1688017	A	0.65	Mature	hsa-mir-6887	hsa-miR-6887-5p
19	35836530	rs10406069	A	0.80	pre-miRNA	hsa-mir-5196	NA
19	40875800	rs3745198	G	0.57	Seed	hsa-mir-6796	hsa-miR-6796-3p
19	40875810	rs3745199	G	0.57	Mature	hsa-mir-6796	hsa-miR-6796-3p
19	47212593	rs10423365	G	0.45	pre-miRNA	hsa-mir-320e	NA
19	50436371	rs8667	A	0.66	pre-miRNA	hsa-mir-4751	NA
19	52725338	rs10412196	C	0.88	Mature	hsa-mir-6801	hsa-miR-6801-3p
19	54185492	rs75598818	A	0.96	pre-miRNA	hsa-mir-520f	NA
19	54191743	rs57111412	G	0.93	pre-miRNA	hsa-mir-1283-1	NA
19	54228742	rs10670323	GAA AGA	0.91	pre-miRNA	hsa-mir-516b-2	NA
19	54238208	rs74704964	T	0.95	pre-miRNA	hsa-mir-518d	NA
19	54245788	rs56013413	A	0.97	pre-miRNA	hsa-mir-520h	NA
19	54261549	rs71363366	G	0.98	pre-miRNA	hsa-mir-1283-2	NA
19	54786022	rs4112253	C	0.63	pre-miRNA	hsa-mir-4752	NA
19	55899602	rs56312243	T	0.94	Mature	hsa-mir-6805	hsa-miR-6805-3p
19	58898176	rs138578962	T	0.98	pre-miRNA	hsa-mir-4754	NA

19	58898193	rs975947	T	0.51	pre-miRNA	hsa-mir-4754	NA
20	7352262	rs73080647	G	0.99	pre-miRNA	hsa-mir-8062	NA
20	18451325	rs11907020	C	0.99	Mature	hsa-mir-3192	hsa-miR-3192-3p
20	33578251	rs3746444	G	0.82	Seed	hsa-mir-499a	hsa-miR-499a-3p
20	57392686	rs117258475	A	0.99	Mature	hsa-mir-296	hsa-miR-296-3p
20	58883534	rs6513496	C	0.77	pre-miRNA	hsa-mir-646	NA
20	58883605	rs6513497	G	0.92	Mature	hsa-mir-646	hsa-miR-646
20	60639909	rs560652437	G	0.90	pre-miRNA	hsa-mir-3195	NA
20	61870141	rs113297757	A	0.98	Seed	hsa-mir-3196	hsa-miR-3196
20	61870167	rs744591	A	0.53	pre-miRNA	hsa-mir-3196	NA
20	62550824	rs2427556	A	0.79	pre-miRNA	hsa-mir-941-1	NA
20	62550965	rs574724312	A	0.99	Seed	hsa-mir-941-3	hsa-miR-941
20	62574006	rs73147065	G	0.83	pre-miRNA	hsa-mir-647	NA
21	44371114	rs451887	C	0.12	Seed	hsa-mir-5692b	hsa-miR-5692b
22	23165340	rs5996397	G	0.83	pre-miRNA	hsa-mir-650	NA
22	26951185	rs4822739	G	0.96	pre-miRNA	hsa-mir-548j	NA
22	28316554	rs576250744	T	0.95	pre-miRNA	hsa-mir-3199-2	NA
22	28316554	rs576250744	T	0.95	pre-miRNA	hsa-mir-3199-1	NA
22	28316560	rs564707204	C	0.95	pre-miRNA	hsa-mir-3199-2	NA
22	28316560	rs564707204	C	0.95	pre-miRNA	hsa-mir-3199-1	NA
22	28316561	rs532189082	A	0.95	pre-miRNA	hsa-mir-3199-2	NA
22	28316561	rs532189082	A	0.95	pre-miRNA	hsa-mir-3199-1	NA
22	28316591	rs75321888	TG	0.90	Mature	hsa-mir-3199-2	hsa-miR-3199
22	28316591	rs75321888	TG	0.90	Mature	hsa-mir-3199-1	hsa-miR-3199
22	31556103	rs5997893	G	0.35	Mature	hsa-mir-3928	hsa-miR-3928-5p
22	35731697	rs9607265	A	0.97	pre-miRNA	hsa-mir-3909	NA
22	38240368	rs141002682	C	0.94	pre-miRNA	hsa-mir-658	NA
22	46156446	rs60308683	A	0.88	pre-miRNA	hsa-mir-4762	NA
22	50356555	rs4078443	C	0.25	pre-miRNA	hsa-mir-6821	NA

EA: effect allele, EAF: effect allele frequency

Table S2. 122 FDR-significant hits in PheWAS

group	description	phenotype	snp	location	pre_id	mature_id	EA	EAF	n_cases	OR(95%CI)	p
circulatory system	Hypertension	401	rs2168518	Seed	hsa-mir-4513	hsa-miR-4513	A	0.68	91878	0.96(0.95-0.98)	8.47E-10
circulatory system	Essential hypertension	401.1	rs2168518	Seed	hsa-mir-4513	hsa-miR-4513	A	0.68	91605	0.96(0.95-0.98)	1.06E-09
circulatory system	Atrial fibrillation and flutter	427.2	rs547611708	pre-miRNA	hsa-mir-1225	NA	TC	0.01	18289	1.32(1.2-1.45)	3.61E-09
circulatory system	Essential hypertension	401.1	rs367805	pre-miRNA	hsa-mir-3936	NA	C	0.7	91605	0.97(0.96-0.98)	1.76E-07
circulatory system	Hypertension	401	rs367805	pre-miRNA	hsa-mir-3936	NA	C	0.7	91878	0.97(0.96-0.98)	1.92E-07
circulatory system	Hypertension	401	rs2070960	Seed	hsa-mir-3620	hsa-miR-3620-5p	T	0.08	91878	0.95(0.93-0.97)	3.70E-07
circulatory system	Essential hypertension	401.1	rs2070960	Seed	hsa-mir-3620	hsa-miR-3620-5p	T	0.08	91605	0.95(0.93-0.97)	5.01E-07
circulatory system	Essential hypertension	401.1	rs3817551	Mature	hsa-mir-7107	hsa-miR-7107-3p	G	0.4	91605	0.97(0.96-0.98)	7.38E-07
circulatory system	Hypertension	401	rs3817551	Mature	hsa-mir-7107	hsa-miR-7107-3p	G	0.4	91878	0.97(0.96-0.98)	1.14E-06
circulatory system	Hypertension	401	rs11382316	Seed	hsa-mir-3161	hsa-miR-3161	TA	0.79	91878	1.03(1.02-1.05)	1.66E-06
circulatory system	Essential hypertension	401.1	rs11382316	Seed	hsa-mir-3161	hsa-miR-3161	TA	0.79	91605	1.03(1.02-1.05)	1.77E-06
circulatory system	Essential hypertension	401.1	rs4687672	Mature	hsa-mir-8064	hsa-miR-8064	A	0.23	91605	1.03(1.02-1.04)	2.65E-06
circulatory system	Hypertension	401	rs4687672	Mature	hsa-mir-8064	hsa-miR-8064	A	0.23	91878	1.03(1.02-1.04)	2.91E-06
circulatory system	Essential hypertension	401.1	rs2925980	Seed	hsa-mir-7854	hsa-miR-7854-3p	G	0.33	91605	0.97(0.96-0.99)	7.03E-06
circulatory system	Varicose veins	454	rs70990234	pre-miRNA	hsa-mir-6891	NA	TGAAGGGCTCCA	0.35	12342	0.94(0.91-0.97)	7.22E-06
circulatory system	Hypertension	401	rs2925980	Seed	hsa-mir-7854	hsa-miR-7854-3p	G	0.33	91878	0.97(0.96-0.99)	7.40E-06
circulatory system	Ischemic Heart Disease	411	rs12456845	pre-miRNA	hsa-mir-4744	NA	C	0.05	35220	1.09(1.05-1.13)	8.39E-06
circulatory system	Varicose veins of lower extremity	454.1	rs70990234	pre-miRNA	hsa-mir-6891	NA	TGAAGGGCTCCA	0.35	11809	0.94(0.91-0.97)	8.98E-06
circulatory system	Essential hypertension	401.1	rs2427556	pre-miRNA	hsa-mir-941-1	NA	A	0.21	91605	1.03(1.02-1.04)	1.01E-05
circulatory system	Hypertension	401	rs2427556	pre-miRNA	hsa-mir-941-1	NA	A	0.21	91878	1.03(1.02-1.04)	1.05E-05
circulatory system	Raynaud's syndrome	443.1	rs67976778	pre-miRNA	hsa-mir-4305	NA	T	0.39	1404	1.18(1.09-1.27)	1.70E-05
congenital anomalies	Other and unspecified congenital anomalies	759	rs374409015	Seed	hsa-mir-4467	hsa-miR-4467	T	0.04	217	2.27(1.58-3.26)	9.34E-06
dermatologic	Psoriasis	696.4	rs70990234	pre-miRNA	hsa-mir-6891	NA	TGAAGGGCTCCA	0.35	2733	1.47(1.39-1.55)	1.28E-43
dermatologic	Psoriasis and related disorders	696	rs70990234	pre-miRNA	hsa-mir-6891	NA	TGAAGGGCTCCA	0.35	2798	1.45(1.38-1.53)	3.05E-42
dermatologic	Psoriasis vulgaris	696.41	rs70990234	pre-miRNA	hsa-mir-6891	NA	TGAAGGGCTCCA	0.35	2062	1.53(1.44-1.63)	1.56E-41

dermatologic	Psoriasis vulgaris	696.41	rs3819276	pre-miRNA	hsa-mir-6891	NA	T	0.01	2062	2.45(2.06-2.91)	2.41E-24
dermatologic	Psoriasis and related disorders	696	rs3819276	pre-miRNA	hsa-mir-6891	NA	T	0.01	2798	2.2(1.88-2.57)	4.34E-23
dermatologic	Psoriasis	696.4	rs3819276	pre-miRNA	hsa-mir-6891	NA	T	0.01	2733	2.2(1.88-2.58)	9.26E-23
dermatologic	Psoriasis and related disorders	696	rs4285314	pre-miRNA	hsa-mir-3135b	NA	A	0.46	2798	1.23(1.16-1.29)	3.43E-14
dermatologic	Psoriasis	696.4	rs4285314	pre-miRNA	hsa-mir-3135b	NA	A	0.46	2733	1.22(1.16-1.29)	1.50E-13
dermatologic	Psoriatic arthropathy	696.42	rs4285314	pre-miRNA	hsa-mir-3135b	NA	A	0.46	849	1.32(1.2-1.45)	1.66E-08
dermatologic	Psoriasis vulgaris	696.41	rs4285314	pre-miRNA	hsa-mir-3135b	NA	A	0.46	2062	1.19(1.12-1.26)	3.58E-08
dermatologic	Psoriatic arthropathy	696.42	rs70990234	pre-miRNA	hsa-mir-6891	NA	TGAAGGGCTCCA	0.35	849	1.31(1.18-1.44)	8.39E-08
dermatologic	Psoriasis vulgaris	696.41	rs2854001	Mature	hsa-mir-6891	hsa-miR-6891-3p	A	0.23	2062	0.81(0.75-0.88)	9.89E-08
dermatologic	Psoriasis	696.4	rs2854001	Mature	hsa-mir-6891	hsa-miR-6891-3p	A	0.23	2733	0.84(0.78-0.9)	1.96E-07
dermatologic	Psoriasis and related disorders	696	rs2854001	Mature	hsa-mir-6891	hsa-miR-6891-3p	A	0.23	2798	0.85(0.8-0.91)	1.06E-06
dermatologic	Actinic keratosis	702.1	rs4285314	pre-miRNA	hsa-mir-3135b	NA	A	0.46	2644	1.14(1.08-1.21)	1.59E-06
dermatologic	Psoriasis and related disorders	696	rs2276448	Seed	hsa-mir-6891	hsa-miR-6891-3p	C	0.14	2798	0.83(0.77-0.9)	6.95E-06
dermatologic	Pruritus and related conditions	698	rs2276448	Seed	hsa-mir-6891	hsa-miR-6891-3p	C	0.14	864	1.32(1.17-1.5)	9.51E-06
dermatologic	Circumscribed scleroderma	701.3	rs17881225	pre-miRNA	hsa-mir-6891	NA	C	0.1	568	1.45(1.23-1.72)	1.45E-05
digestive	Celiac disease	557.1	rs4285314	pre-miRNA	hsa-mir-3135b	NA	A	0.46	2079	0.37(0.35-0.4)	1.80E-162
digestive	Celiac disease	557.1	rs70990234	pre-miRNA	hsa-mir-6891	NA	TGAAGGGCTCCA	0.35	2079	0.5(0.47-0.54)	3.59E-72
digestive	Celiac disease	557.1	rs2276448	Seed	hsa-mir-6891	hsa-miR-6891-3p	C	0.14	2079	0.5(0.44-0.56)	1.73E-32
digestive	Celiac disease	557.1	rs2854001	Mature	hsa-mir-6891	hsa-miR-6891-3p	A	0.23	2079	0.69(0.64-0.75)	2.09E-19
digestive	Celiac disease	557.1	rs4351242	pre-miRNA	hsa-mir-3135b	NA	T	0.08	2079	1.52(1.38-1.66)	5.39E-18
digestive	Celiac disease	557.1	rs17881225	pre-miRNA	hsa-mir-6891	NA	C	0.1	2079	0.64(0.57-0.72)	1.23E-12
digestive	Cholelithiasis	574.1	rs174561	pre-miRNA	hsa-mir-1908	NA	C	0.31	15056	1.07(1.04-1.1)	4.93E-08
digestive	Cholelithiasis and cholecystitis	574	rs174561	pre-miRNA	hsa-mir-1908	NA	C	0.31	16867	1.06(1.04-1.09)	8.37E-07
digestive	Reflux esophagitis	530.14	rs11614913	Mature	hsa-mir-196a-2	hsa-miR-196a-3p	T	0.42	12091	1.06(1.04-1.09)	3.32E-06
digestive	Hepatomegaly	573.3	rs72563729	pre-miRNA	hsa-mir-200b	NA	A	0.01	305	2.9(1.83-4.59)	6.06E-06
digestive	Celiac disease	557.1	rs3819276	pre-miRNA	hsa-mir-6891	NA	T	0.01	2079	0.4(0.27-0.61)	1.41E-05

endocrine/metabolic	Hypothyroidism NOS	244.4	rs17881225	pre-miRNA	hsa-mir-6891	NA	C	0.1	17127	0.83(0.8-0.86)	2.99E-21
endocrine/metabolic	Hypothyroidism	244	rs17881225	pre-miRNA	hsa-mir-6891	NA	C	0.1	17960	0.83(0.8-0.86)	3.43E-21
endocrine/metabolic	Type 1 diabetes	250.1	rs4285314	pre-miRNA	hsa-mir-3135b	NA	A	0.46	2840	0.78(0.74-0.82)	2.50E-20
endocrine/metabolic	Type 1 diabetes	250.1	rs2276448	Seed	hsa-mir-6891	hsa-miR-6891-3p	C	0.14	2840	0.74(0.68-0.81)	3.55E-12
endocrine/metabolic	Type 1 diabetes with ketoacidosis	250.11	rs4285314	pre-miRNA	hsa-mir-3135b	NA	A	0.46	271	0.52(0.44-0.63)	4.62E-12
endocrine/metabolic	Type 1 diabetes	250.1	rs17881225	pre-miRNA	hsa-mir-6891	NA	C	0.1	2840	1.3(1.21-1.41)	3.90E-11
endocrine/metabolic	Hypothyroidism	244	rs368791729	Mature	hsa-mir-3939	hsa-miR-3939	G	0.38	17960	1.08(1.06-1.11)	8.06E-11
endocrine/metabolic	Hypothyroidism	244	rs370955537	Mature	hsa-mir-3939	hsa-miR-3939	C	0.38	17960	1.08(1.06-1.11)	8.06E-11
endocrine/metabolic	Hypothyroidism NOS	244.4	rs368791729	Mature	hsa-mir-3939	hsa-miR-3939	G	0.38	17127	1.08(1.05-1.11)	4.17E-10
endocrine/metabolic	Hypothyroidism NOS	244.4	rs370955537	Mature	hsa-mir-3939	hsa-miR-3939	C	0.38	17127	1.08(1.05-1.11)	4.17E-10
endocrine/metabolic	Diabetes mellitus	250	rs11435035	pre-miRNA	hsa-mir-5094	NA	CT	0.7	22996	0.94(0.92-0.96)	8.60E-09
endocrine/metabolic	Type 2 diabetes	250.2	rs11435035	pre-miRNA	hsa-mir-5094	NA	CT	0.7	21947	0.94(0.92-0.96)	1.20E-08
endocrine/metabolic	Hypothyroidism NOS	244.4	rs70990234	pre-miRNA	hsa-mir-6891	NA	TGAAGGGCTCCA	0.35	17127	0.94(0.91-0.96)	3.11E-08
endocrine/metabolic	Hypothyroidism	244	rs70990234	pre-miRNA	hsa-mir-6891	NA	TGAAGGGCTCCA	0.35	17960	0.94(0.92-0.96)	1.17E-07
endocrine/metabolic	Hypoglycemia	251.1	rs4285314	pre-miRNA	hsa-mir-3135b	NA	A	0.46	1151	0.8(0.74-0.87)	1.78E-07
endocrine/metabolic	Diabetes mellitus	250	rs243080	pre-miRNA	hsa-mir-4432	NA	A	0.45	22996	0.95(0.93-0.97)	5.68E-07
endocrine/metabolic	Hypercholesterolemia	272.11	rs1003723	pre-miRNA	hsa-mir-6886	NA	T	0.46	39763	1.04(1.02-1.05)	6.65E-07
endocrine/metabolic	Hypothyroidism	244	rs174561	pre-miRNA	hsa-mir-1908	NA	C	0.31	17960	0.94(0.92-0.96)	6.75E-07
endocrine/metabolic	Disorders of lipoid metabolism	272	rs1003723	pre-miRNA	hsa-mir-6886	NA	T	0.46	42979	1.04(1.02-1.05)	9.05E-07
endocrine/metabolic	Hyperlipidemia	272.1	rs1003723	pre-miRNA	hsa-mir-6886	NA	T	0.46	42854	1.04(1.02-1.05)	1.01E-06
endocrine/metabolic	Hypothyroidism NOS	244.4	rs174561	pre-miRNA	hsa-mir-1908	NA	C	0.31	17127	0.94(0.92-0.97)	1.58E-06
endocrine/metabolic	Obesity	278.1	rs897984	pre-miRNA	hsa-mir-4519	NA	C	0.61	15317	0.95(0.92-0.97)	2.03E-06
endocrine/metabolic	Diabetes mellitus	250	rs4687672	Mature	hsa-mir-8064	hsa-miR-8064	A	0.23	22996	1.06(1.03-1.08)	2.14E-06
endocrine/metabolic	Thyrotoxicosis with or without goiter	242	rs2854001	Mature	hsa-mir-6891	hsa-miR-6891-3p	A	0.23	2163	0.83(0.77-0.9)	2.45E-06
endocrine/metabolic	Type 2 diabetes	250.2	rs243080	pre-miRNA	hsa-mir-4432	NA	A	0.45	21947	0.95(0.94-0.97)	3.07E-06
endocrine/metabolic	Hypoglycemia	251.1	rs117428639	pre-miRNA	hsa-mir-2113	NA	T	0.02	1151	1.73(1.37-2.19)	3.44E-06
endocrine/metabolic	Overweight, obesity and other hyperalimentation	278	rs897984	pre-miRNA	hsa-mir-4519	NA	C	0.61	15480	0.95(0.92-0.97)	3.56E-06
endocrine/metabolic	Type 2 diabetes	250.2	rs4687672	Mature	hsa-mir-8064	hsa-miR-8064	A	0.23	21947	1.05(1.03-1.08)	4.78E-06
endocrine/metabolic	Hyperlipidemia	272.1	rs2276448	Seed	hsa-mir-6891	hsa-miR-6891-3p	C	0.14	42854	1.05(1.03-1.07)	1.01E-05

endocrine/metabolic	Hypercholesterolemia	272.11	rs2276448	Seed	hsa-mir-6891	hsa-miR-6891-3p	C	0.14	39763	1.05(1.03-1.07)	1.29E-05
endocrine/metabolic	Diabetes mellitus	250	rs4078443	pre-miRNA	hsa-mir-6821	NA	C	0.75	22996	0.95(0.93-0.97)	1.53E-05
endocrine/metabolic	Disorders of lipoid metabolism	272	rs2276448	Seed	hsa-mir-6891	hsa-miR-6891-3p	C	0.14	42979	1.05(1.02-1.07)	1.69E-05
endocrine/metabolic	Vitamin B-complex deficiencies	261.2	rs2276448	Seed	hsa-mir-6891	hsa-miR-6891-3p	C	0.14	1049	0.74(0.64-0.85)	1.77E-05
endocrine/metabolic	Type 2 diabetes	250.2	rs4078443	pre-miRNA	hsa-mir-6821	NA	C	0.75	21947	0.95(0.93-0.97)	1.90E-05
genitourinary	Hematuria	593	rs70990234	pre-miRNA	hsa-mir-6891	NA	TGAAGGGCTCCA	0.35	16317	1.07(1.05-1.1)	5.71E-09
genitourinary	Hematuria	593	rs4285314	pre-miRNA	hsa-mir-3135b	NA	A	0.46	16317	1.06(1.04-1.08)	2.36E-07
infectious diseases	Dermatophytosis	110.1	rs35854553	pre-miRNA	hsa-mir-3166	NA	A	0.09	297	1.71(1.37-2.14)	2.56E-06
injuries & poisonings	Allergy/adverse effect of penicillin	960.2	rs2276448	Seed	hsa-mir-6891	hsa-miR-6891-3p	C	0.14	18091	1.08(1.05-1.12)	1.20E-07
injuries & poisonings	Poisoning by antibiotics	960	rs2276448	Seed	hsa-mir-6891	hsa-miR-6891-3p	C	0.14	20790	1.08(1.05-1.11)	1.54E-07
mental disorders	Aphasia/speech disturbance	292.1	rs11713052	pre-miRNA	hsa-mir-5092	NA	G	0.03	1016	1.84(1.49-2.27)	1.33E-08
mental disorders	Anxiety disorders	300	rs67106263	Mature	hsa-mir-3144	hsa-miR-3144-3p	A	0.24	8627	0.92(0.89-0.95)	5.38E-06
mental disorders	Anxiety disorders	300	rs68035463	pre-miRNA	hsa-mir-3144	NA	A	0.24	8627	0.92(0.89-0.95)	5.57E-06
mental disorders	Anxiety disorder	300.1	rs67106263	Mature	hsa-mir-3144	hsa-miR-3144-3p	A	0.24	7962	0.92(0.89-0.96)	1.57E-05
mental disorders	Anxiety disorder	300.1	rs68035463	pre-miRNA	hsa-mir-3144	NA	A	0.24	7962	0.92(0.89-0.96)	1.60E-05
musculoskeletal	Ankylosing spondylitis	715.2	rs2854001	Mature	hsa-mir-6891	hsa-miR-6891-3p	A	0.23	467	3.23(2.84-3.67)	2.53E-71
musculoskeletal	Other inflammatory spondylopathies	715	rs2854001	Mature	hsa-mir-6891	hsa-miR-6891-3p	A	0.23	1821	1.49(1.39-1.6)	3.46E-28
musculoskeletal	Rheumatoid arthritis	714.1	rs4351242	pre-miRNA	hsa-mir-3135b	NA	T	0.08	4863	0.71(0.65-0.77)	1.26E-15
musculoskeletal	Rheumatoid arthritis and other inflammatory polyarthropathies	714	rs4351242	pre-miRNA	hsa-mir-3135b	NA	T	0.08	5494	0.73(0.68-0.79)	7.24E-15
musculoskeletal	Ankylosing spondylitis	715.2	rs70990234	pre-miRNA	hsa-mir-6891	NA	TGAAGGGCTCCA	0.35	467	0.57(0.49-0.67)	7.22E-13
musculoskeletal	Rheumatoid arthritis and other inflammatory polyarthropathies	714	rs4285314	pre-miRNA	hsa-mir-3135b	NA	A	0.46	5494	1.13(1.09-1.17)	2.45E-10
musculoskeletal	Rheumatoid arthritis	714.1	rs4285314	pre-miRNA	hsa-mir-3135b	NA	A	0.46	4863	1.13(1.09-1.18)	7.21E-10
musculoskeletal	Contracture of palmar fascia [Dupuytren's disease]	728.71	rs60308683	pre-miRNA	hsa-mir-4762	NA	A	0.12	3900	1.21(1.13-1.29)	1.96E-08
musculoskeletal	Fasciitis	728.7	rs60308683	pre-miRNA	hsa-mir-4762	NA	A	0.12	4276	1.19(1.12-1.27)	4.14E-08

musculoskeletal	Ankylosing spondylitis	715.2	rs2276448	Seed	hsa-mir-6891	hsa-miR-6891-3p	C	0.14	467	0.57(0.45-0.71)	1.23E-06
musculoskeletal	Intervertebral disc disorders	722	rs2168518	Seed	hsa-mir-4513	hsa-miR-4513	A	0.68	1189	1.25(1.14-1.37)	1.56E-06
musculoskeletal	Polymyalgia Rheumatica	717	rs4351242	pre-miRNA	hsa-mir-3135b	NA	T	0.08	1535	0.7(0.6-0.81)	2.93E-06
musculoskeletal	Spinal stenosis	720	rs13299349	Mature	hsa-mir-3152	hsa-miR-3152-3p	A	0.32	4713	0.9(0.86-0.94)	7.62E-06
musculoskeletal	Osteoarthritis; localized	740.1	rs2168518	Seed	hsa-mir-4513	hsa-miR-4513	A	0.68	36573	1.04(1.02-1.06)	1.08E-05
musculoskeletal	Other and unspecified disc disorder	722.9	rs2168518	Seed	hsa-mir-4513	hsa-miR-4513	A	0.68	907	1.26(1.14-1.4)	1.21E-05
neoplasms	Benign neoplasm of colon	208	rs174561	pre-miRNA	hsa-mir-1908	NA	C	0.31	25561	0.95(0.93-0.96)	3.19E-08
neoplasms	Nodular lymphoma	202.21	rs11435035	pre-miRNA	hsa-mir-5094	NA	CT	0.7	439	0.73(0.63-0.84)	7.75E-06
neoplasms	Malignant neoplasm of rectum, rectosigmoid junction, and anus	153.3	rs2276448	Seed	hsa-mir-6891	hsa-miR-6891-3p	C	0.14	2528	0.83(0.76-0.9)	1.48E-05
neurological	Multiple sclerosis	335	rs4285314	pre-miRNA	hsa-mir-3135b	NA	A	0.46	1462	0.75(0.69-0.8)	1.63E-14
neurological	Multiple sclerosis	335	rs2854001	Mature	hsa-mir-6891	hsa-miR-6891-3p	A	0.23	1462	0.69(0.63-0.76)	1.23E-13
neurological	Anterior horn cell disease	334.2	rs117723462	pre-miRNA	hsa-mir-3649	NA	G	0.02	310	2.49(1.66-3.71)	8.61E-06
respiratory	Influenza	481	rs140379047	Mature	hsa-mir-585	hsa-miR-585-5p	G	0.01	361	2.6(1.75-3.85)	2.11E-06
respiratory	Nasal polyps	471	rs174561	pre-miRNA	hsa-mir-1908	NA	C	0.31	3501	0.88(0.84-0.93)	3.43E-06
respiratory	Chronic tonsillitis and adenoiditis	474.2	rs5996397	pre-miRNA	hsa-mir-650	NA	G	0.17	1176	1.26(1.14-1.39)	6.88E-06
respiratory	Pneumonitis due to inhalation of food or vomitus	501	rs146892675	pre-miRNA	hsa-mir-199b	NA	T	0.01	809	2.17(1.52-3.09)	1.72E-05
sense organs	Myopia	367.1	rs35196866	pre-miRNA	hsa-mir-4669	NA	A	0.76	1739	1.21(1.11-1.31)	7.69E-06
sense organs	Hearing loss	389	rs12803915	pre-miRNA	hsa-mir-612	NA	A	0.22	5736	0.9(0.86-0.94)	8.84E-06

EA: effect allele, EAF: effect allele frequency, OR: odds ratio, CI: confidence interval.

Table S3. Candidate target genes whose interaction might be implicated by the presence of SNPs in seed region of miRNAs.

Gene_name	trait	miRNA	MTI
PHF19	PP	hsa-miR-3620-5p	validated
SLC7A1	DBP	hsa-miR-3620-5p	validated
SLC7A1	SBP	hsa-miR-3620-5p	validated
RELA	SBP	hsa-miR-3161	validated
SLC8A1	SBP	hsa-miR-3161	validated
TERT	SBP	hsa-miR-3161	validated
CELF1	SBP	hsa-miR-7854-3p	validated
HOXB6	SBP	hsa-miR-7854-3p	validated
HOXB6	PP	hsa-miR-7854-3p	validated
MAPKAPK5	SBP	hsa-miR-7854-3p	validated
MAPKAPK5	DBP	hsa-miR-7854-3p	validated
OTUB1	PP	hsa-miR-7854-3p	validated
OVOL1	SBP	hsa-miR-7854-3p	validated
PHACTR4	SBP	hsa-miR-7854-3p	validated
VLDLR	LDL	hsa-miR-6891-3p	validated
VLDLR	TC	hsa-miR-6891-3p	validated

MTI: miRNA and target gene interaction
PP: pulse pressure
DBP: diastolic blood pressure
SBP: systolic blood pressure
LDL: low density lipoprotein cholesterol
TC: total cholesterol

Table S4. Genetic variants in miRNAs that affect plasma levels of corresponding mature miRNAs (P<0.05).

SNP	miRNA	location	EA	EAF	beta	SE	P	Study
rs10934682	miR-544b	pre-miRNA	G	0.18	0.09	0.03	1.72E-03	(25)
rs117650137	miR-6717-5p	Seed	G	0.97	0.26	0.10	1.15E-02	(24)
rs12416605	miR-938	Seed	C	0.74	0.11	0.05	2.79E-02	(24)
rs12512664	miR-4274	pre-miRNA	A	0.58	-0.02	0.01	2.17E-02	(24)
rs12803915	miR-612	pre-miRNA	G	0.77	0.07	0.03	3.87E-02	(24)
rs138552125	miR-8070	Seed	C	0.98	0.27	0.12	2.34E-02	(24)
rs1514422	miR-8060	Mature	G	0.95	0.21	0.08	5.54E-03	(24)
rs17091403	miR-2110	pre-miRNA	C	0.88	0.14	0.03	6.60E-07	(24)
rs17091403	miR-2110	pre-miRNA	T	0.07	-0.10	0.02	9.90E-06	(25)
rs174561	miR-1908-5p	pre-miRNA	T	0.70	-0.10	0.02	7.66E-08	(24)
rs174561	miR-1908-5p	pre-miRNA	C	0.30	0.15	0.01	4.76E-31	(25)
rs2114358	miR-1206	pre-miRNA	G	0.38	-0.10	0.04	2.93E-02	(24)
rs2168518	miR-4513	Seed	G	0.32	0.08	0.04	4.36E-02	(24)
rs2241347	miR-3130-3p	Mature	C	0.84	-0.27	0.03	5.49E-16	(24)
rs2241347	miR-3130-3p	Mature	T	0.18	0.17	0.03	1.18E-07	(25)
rs2273626	miR-4707-3p	Seed	A	0.49	0.09	0.01	3.41E-11	(24)
rs2276448	miR-6891-3p	Seed	T	0.75	0.13	0.03	2.39E-06	(24)
rs257095	miR-4636	pre-miRNA	C	0.13	0.13	0.06	3.32E-02	(24)
rs2682818	miR-618	pre-miRNA	A	0.13	-0.23	0.05	1.35E-05	(24)
rs2854001	miR-6891-3p	Mature	G	0.85	0.17	0.04	1.12E-05	(24)
rs2925980	miR-7854-3p	Seed	G	0.32	-0.06	0.02	1.62E-02	(25)
rs2967897	miR-5695	pre-miRNA	T	0.38	0.09	0.03	1.60E-03	(25)
rs4078443	miR-6821-5p	pre-miRNA	T	0.27	-0.05	0.01	7.30E-04	(25)
rs41291179	miR-216a-5p	pre-miRNA	A	0.95	-0.15	0.06	6.84E-03	(24)
rs45596840	miR-4482-5p	Seed	G	0.69	0.21	0.04	9.33E-07	(24)
rs550894	miR-612	pre-miRNA	C	0.91	0.11	0.05	2.71E-02	(24)
rs56103835	miR-323b-3p	pre-miRNA	T	0.79	0.17	0.03	5.90E-07	(24)
rs56103835	miR-323b-3p	pre-miRNA	C	0.22	-0.26	0.02	1.20E-26	(25)
rs56148568	miR-7157-3p	Mature	T	0.78	0.14	0.05	1.81E-03	(24)
rs56195815	miR-548aw	pre-miRNA	T	0.21	-0.05	0.03	4.18E-02	(25)
rs58605477	miR-7150	pre-miRNA	A	0.78	0.04	0.02	1.22E-02	(24)
rs619608	miR-6726-3p	Seed	A	0.04	-0.14	0.05	6.52E-03	(25)
rs62154973	miR-4772-5p	Mature	C	0.90	0.19	0.06	2.63E-03	(24)
rs641071	miR-4482-5p	pre-miRNA	G	0.64	0.16	0.04	1.23E-04	(24)
rs6943868	miR-3683	pre-miRNA	A	0.03	-0.21	0.09	1.76E-02	(25)
rs7207008	miR-2117	pre-miRNA	A	0.54	0.06	0.03	4.13E-02	(24)
rs7208391	miR-6868-3p	Mature	C	0.57	0.15	0.04	1.88E-04	(24)
rs72502717	miR-3689f	pre-miRNA	A	0.84	-0.11	0.05	2.07E-02	(24)
rs72810954	miR-4679	Mature	G	0.98	-0.39	0.15	1.23E-02	(24)
rs73239138	miR-1269a	Mature	G	0.76	0.07	0.04	4.87E-02	(24)
rs74469188	miR-6504-5p	Mature	C	0.12	-0.08	0.04	3.04E-02	(25)
rs76347846	miR-6841-3p	Mature	A	0.94	0.16	0.05	6.04E-04	(24)

rs76481776	miR-182-5p	pre-miRNA	C	0.89	-0.10	0.04	7.95E-03	(24)
rs7804972	miR-6839-5p	Mature	G	0.60	0.14	0.04	5.53E-05	(24)
rs78212770	miR-629-5p	Mature	C	0.99	0.36	0.10	3.75E-04	(24)
rs7911488	miR-1307-5p	pre-miRNA	A	0.68	0.04	0.02	1.63E-02	(24)
rs7911488	miR-1307-3p	pre-miRNA	G	0.32	-0.04	0.01	4.08E-04	(25)
rs7911488	miR-1307-5p	pre-miRNA	G	0.32	-0.07	0.01	6.98E-12	(25)
rs79512808	miR-3977	pre-miRNA	T	0.99	0.97	0.32	1.99E-03	(24)
rs80128580	miR-5707	pre-miRNA	G	0.96	-0.35	0.12	2.53E-03	(24)
rs895819	miR-27a-5p	pre-miRNA	T	0.67	0.12	0.03	2.04E-05	(24)
rs2910164	miR-146a-3p	Seed	C	0.24	0.07	0.02	1.14E-03	(25)

EA: effect allele, EAF: effect allele frequency, SE: standard error

Table S5. Results of colocalisation analysis

nsnps	PP H0	PP H1	PP H2	PP H3	PP H4	trait	shared variant	SNP PP H4
miR-6891-3p								
73	$3.33x10^{-7}$	$4.94\ x10^{-7}$	$3.47\ x10^{-3}$	$4.15\ x10^{-3}$	0.99	TC	rs2596501	0.99
73	$1.03x10^{-8}$	$1.53\ x10^{-8}$	$1.38\ x10^{-1}$	$2.04\ x10^{-1}$	0.66	TG	rs3130614	0.99
73	$1.54x10^{-1}$	$2.29\ x10^{-1}$	$3.09\ x10^{-3}$	$3.97\ x10^{-3}$	0.61	HDL-C	rs2596501	0.99
73	$2.43x10^{-2}$	$3.60\ x10^{-2}$	$2.21\ x10^{-1}$	$3.28\ x10^{-1}$	0.39	LDL-C	rs2596501	0.99
199	$9.8x10^{-97}$	$1.57\ x10^{-96}$	$3.74\ x10^{-1}$	$5.97\ x10^{-1}$	0.03	T1D	rs2855812	0.99
miR-6821-5p								
826	$7.12x10^{-5}$	$1.23x10^{-6}$	$9.71x10^{-1}$	$1.67x10^{-2}$	0.01	T2D	rs36155743	0.15

nsnps: number of SNPs in the region used for colocalisation
TC: total cholesterol; TG: triglycerides; HDL-C: high-density lipoprotein cholesterol; LDL-C: low density lipoprotein cholesterol; T1D: type 1 diabetes; T2D: type 2 diabetes
PP: posterior probability of H0-H4 being true
H0: neither trait has a genetic association in the region; H1: only miRNA has a genetic association in the region; H2: only the disease has a genetic association in the region; H3: both traits are associated, but with different causal variants; H4: both traits are associated and share a single causal variant; SNP.PP.H4: posterior probability that the SNP being causal conditional on H4 being true. Shared variant corresponds to SNP with the highest PP H4.

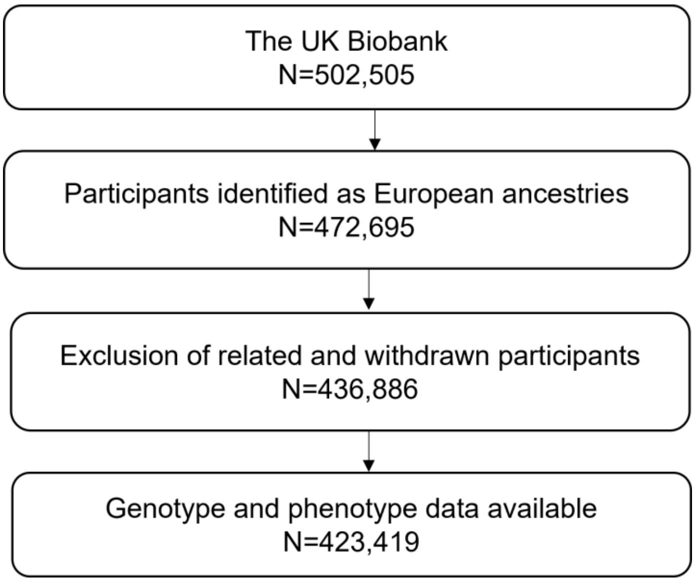

Fig. S1. Selection of study participants.

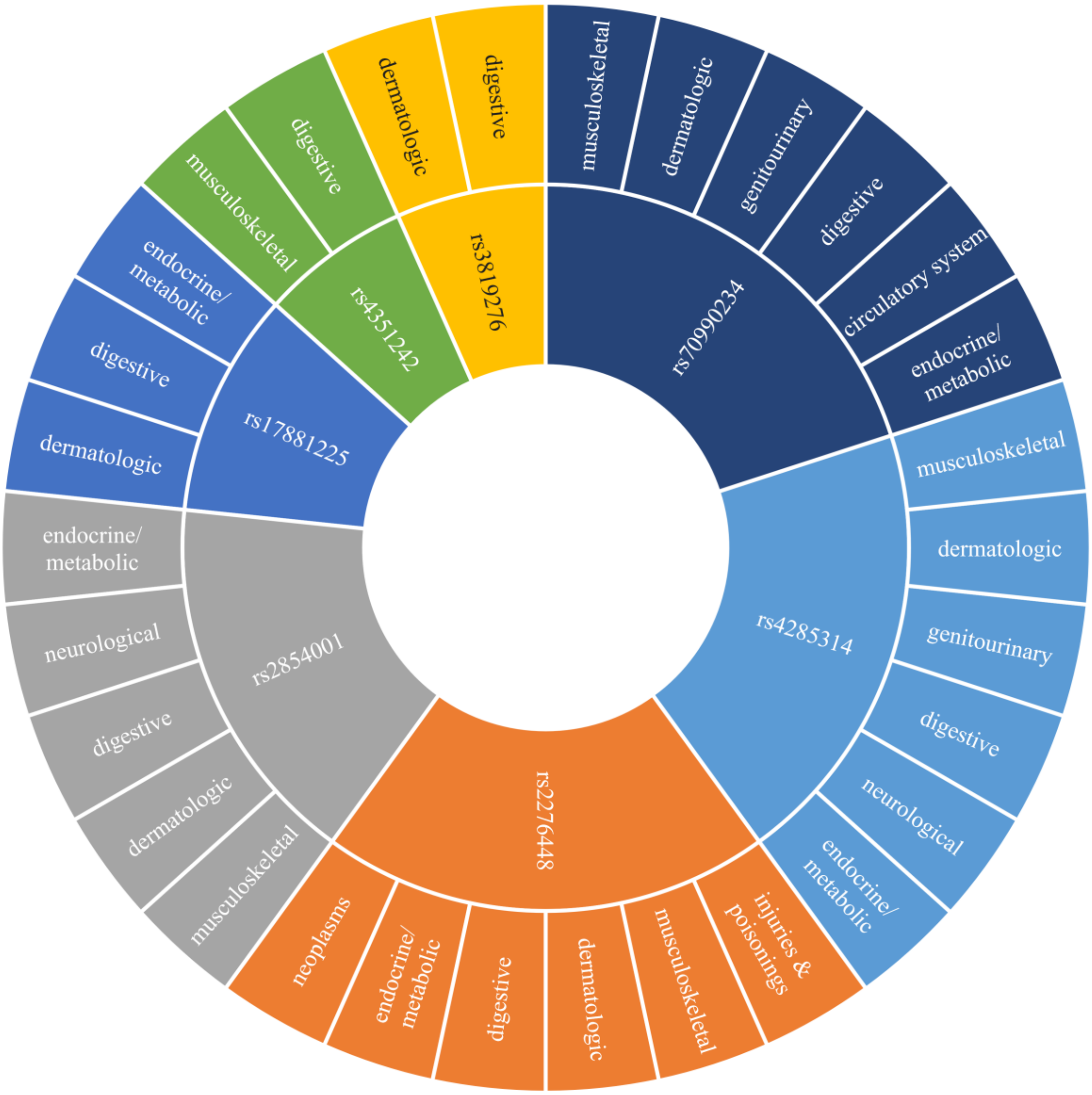

Fig. S2. Each tested variant in MHC is associated with multiple disease groups.

a.

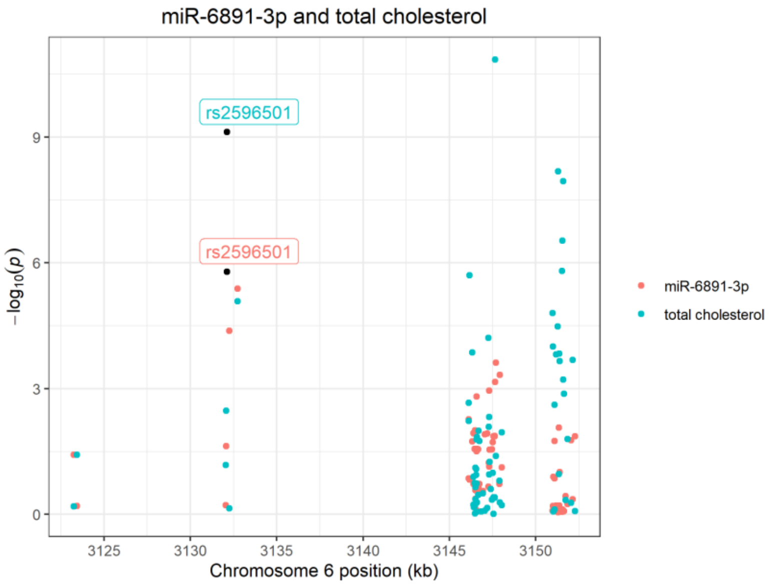

c.

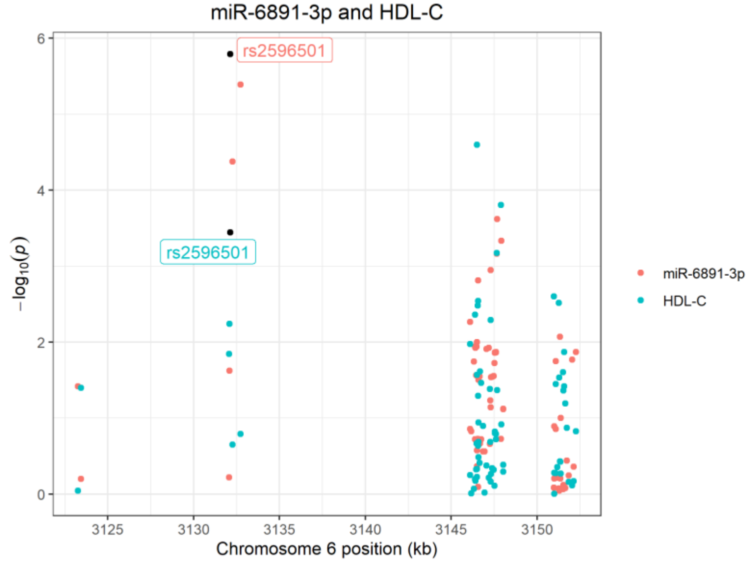

b.

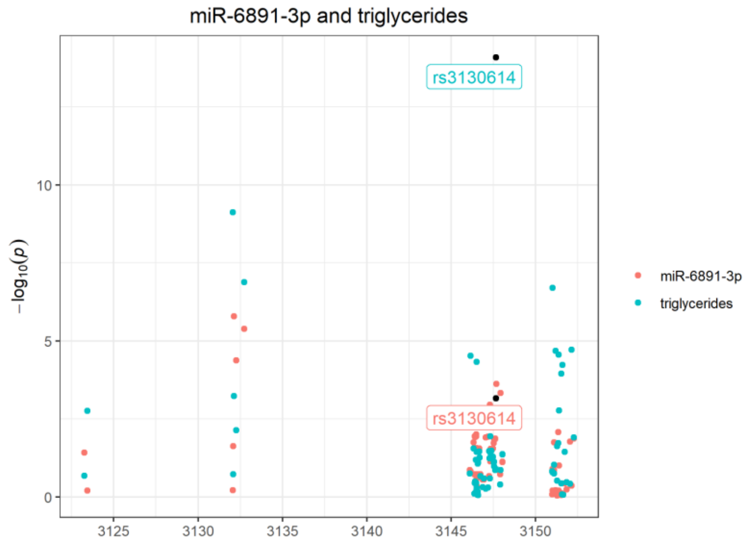

Fig. S3. Regional plot for colocalisation analysis between miR-6891-3p and total cholesterol (a), triglycerides (b), and high-density lipoprotein cholesterol (HDL-C). Variants were filtered based on imputation quality (Rsq>0.7). The most likely shared causal variant is labelled.
